# Structure-based redesign of docking domain interactions modulates the product spectrum of a rhabdopeptide-synthesizing NRPS

**DOI:** 10.1038/s41467-018-06712-1

**Published:** 2018-10-19

**Authors:** Carolin Hacker, Xiaofeng Cai, Carsten Kegler, Lei Zhao, A. Katharina Weickhmann, Jan Philip Wurm, Helge B. Bode, Jens Wöhnert

**Affiliations:** 1 Institute of Molecular Biosciences and Center for Biomolecular Magnetic Resonance (BMRZ), Goethe University Frankfurt, 60438 Frankfurt am Main, Germany; 2 Molecular Biotechnology, Department of Biosciences, Goethe University Frankfurt, 60438 Frankfurt am Main, Germany; 3 Institute of Biophysics and Physical Biochemistry, University of Regensburg, 93053 Regensburg, Germany; 4 Buchmann Institute for Molecular Life Sciences (BMLS), Goethe University Frankfurt, 60438 Frankfurt am Main, Germany

## Abstract

Several peptides in clinical use are derived from non-ribosomal peptide synthetases (NRPS). In these systems multiple NRPS subunits interact with each other in a specific linear order mediated by specific docking domains (DDs), whose structures are not known yet, to synthesize well-defined peptide products. In contrast to classical NRPSs, single-module NRPS subunits responsible for the generation of rhabdopeptide/xenortide-like peptides (RXPs) can act in different order depending on subunit stoichiometry thereby producing peptide libraries. To define the basis for their unusual interaction patterns, we determine the structures of all N-terminal DDs (^N^DDs) as well as of an ^N^DD-^C^DD complex and characterize all putative DD interactions thermodynamically for such a system. Key amino acid residues for DD interactions are identified that upon their exchange change the DD affinity and result in predictable changes in peptide production. Recognition rules for DD interactions are identified that also operate in other megasynthase complexes.

## Abstract (web summary)

Rhabdopeptides are synthesized by non-ribosomal peptide synthetases (NRPSs) and the multiple NRPS subunits interact through docking domains (DD). Here the authors provide insights into DD interaction patterns and present the structures of three N-terminal docking domains (^N^DD) and a ^N^DD-^C^DD complex and derive a set of recognition rules for DD interactions.

## Introduction

Non-ribosomal peptides are a large family of structurally diverse and pharmacologically useful natural products with broad biological activities. Prominent examples are the antibiotic daptomycin^1^ or the immunosuppressant cyclosporine A^2^. They are assembled by multifunctional enzyme complexes called non-ribosomal peptide synthetases (NRPSs) that are organized in a modular fashion. Each module activates and modifies a specific amino acid (aa) that is then subsequently elongated with an aa activated and modified by the next module thereby generating peptides with their length depending on the number of modules used. A typical NRPS elongation module consists of an adenylation (A) domain for activation of a specific aa as aminoacyl adenylate, a condensation (C) domain for peptide bond formation, and a thiolation (T) domain. In the T domains, the aas are covalently attached as reactive thioesters to a phosphopantetheine arm for transfer to the next module^3,4^. In classical NRPSs, different subunits (i.e., individual NRPS proteins often containing multiple modules on a single protein chain) selectively interact with each other non-covalently in a strictly defined order following the collinearity rule and give rise to the synthesis of peptides with defined sequences. Non-covalent interactions between NRPS subunits are mediated by specialized N- and C-terminal docking domains (DDs). Stachelhaus and co-workers have demonstrated that for the NRPS systems synthesizing tyrocidin and surfactin matching pairs of short DD or COM (communication-mediating) domains at the C-terminus of the peptidyl-donating NRPS (^C^DD, ~25 aas) and the N-terminus of the accepting NRPS (^N^DD, ~14 aas) play a decisive role in defining the order of interactions between subunits in vitro and in vivo by swapping COM domains between different subunits^5^. They also constructed a “universal COM system” in vitro by a comparison of COM domains in the tyrocidin A and surfactin-like NRPSs, which led to enzyme crosstalk between different biosynthetic systems that promoted the combinatorial biosynthesis of different peptides^6,7^. The structures of these DDs have not been elucidated so far but interactions between DDs have been mapped based on photocrosslinking experiments^8^. However, for polyketide synthase (PKS) systems that are also organized as modular megasynthases, three structurally different types of DD pairs have been described so far and two structures for ^N^DDs without a bound ^C^DD have been solved for NRPS/PKS hybrid systems^9–14^.

**Fig. 1:**
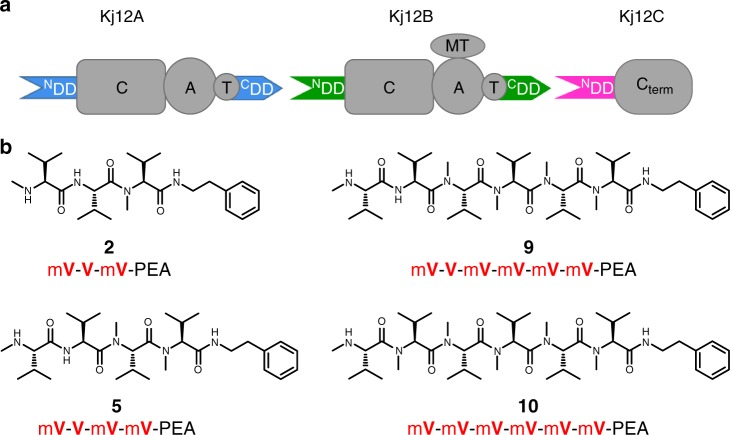
RXP NRPSs (Kj12ABC) from *Xenorhabdus stockiae* KJ12.1 and selected RXPs found in this strain. **a** Overview of the domain organization of Kj12ABC (C condensation, A adenylation, MT methyltransferase, T thiolation, C_term_ terminal condensation domain, ^N^DD N-terminal docking domain, ^C^DD C-terminal docking domain). **b** Structures of selected RXPs derived from the Kj12ABC system, showing differences in size and methylation patterns. The simplified structure nomenclature used within other figures is also shown (V Val, mV *N*-methylated Val, PEA phenylethylamine)

Classic NRPSs are often multimodular—each protein subunit normally consists of the processing modules for multiple aas arranged in a linear fashion. Furthermore, the different protein subunits (i.e., multiple NRPS subunits representing the complete assembly line) interact with each other in a strictly defined linear order yielding a single peptide product with a sequence that faithfully reproduces the linear order of the processing modules along the protein chains. A novel class of NRPSs where up to three single-module NRPS subunits produce complex libraries of rhabdopeptide/xenortide-like peptides (RXPs) has been recently described from entomopathogenic bacteria of the genera *Xenorhabdus* and *Photorhabdus*^15^. The RXP product spectra of different strains differ mainly in peptide length and sequence. In order to synthesize such a range of related products by a single NRPS system, an iterative use of certain NRPS modules or the activity of multiple copies of these modules is apparently required. The observed product spectrum also depends on the stoichiometry of NRPS modules acting in elongation and termination (Supplementary Fig. 1)^15^. This suggests that the subunits of these NRPSs interact with each other not in a well-defined linear order but stochastically. Furthermore, the individual single-module subunits of these NRPSs contain putative N- and C-terminal DDs (^N^CC and ^C^DD, respectively) that differ in length from and show no sequence homology to the DDs in the well-characterized tyrocidin and surfactin producing NRPSs. The Kj12ABC NRPS system from *Xenorhabdus stockiae* KJ12.1 consisting of the three proteins Kj12A, Kj12B, and Kj12C (Fig. 1) is an example for an RXP-synthesizing NRPS. The peptide spectrum produced by this NRPS includes peptides with 2–8 valine and *N-*methyl valine residues in varying orders. The A domains in Kj12A and Kj12B are both specific for Val. However, module Kj12B includes an additional methyltransferase domain that further modifies valine to *N-*methyl valine. While Kj12A and Kj12B are single-module NRPS subunits, Kj12C is a stand-alone C domain that transfers the final peptide chain bound to the phosphopantetheine arm of the T domain in Kj12B to a free amine, mostly phenylethylamine (PEA) (Fig. 1a, Supplementary Fig. 1)^15^. However, the sequences of the RXP products are biased suggesting that some subunits in this system are used preferentially. These preferences might reflect differences in the interactions between subunits as mediated by their DDs. The structural and thermodynamic basis for the differential DD interactions in these unusual NRPS systems and in NRPS systems in general is not clear. Therefore, we characterize DD interactions in these systems in detail and compare them to DD interactions from classical NRPS and other NRPS/PKS systems. We determine the structures of all ^N^DDs in the three protein NRPS system Kj12ABC from *X. stockiae* KJ12.1^15^. We also characterize the thermodynamic basis for the interaction of these ^N^DDs with the two ^C^DDs present in this system and solve the structure of one ^N^DD–^C^DD complex. The structural information for the ^N^DD/^C^DD interaction allows us to derive a set of simple recognition rules for this type of DD interactions as well as the targeted reprogramming of selected DDs via rationally designed aa exchanges leading to differences in the produced peptides. The type of ^N^DD/^C^DD interaction observed for this NRPS system is also found in other megasynthase systems from widely divergent classes of bacteria.

## Results

### Structure determination of ^N^DDs in RXP-type NRPS subunits

**Fig. 2:**
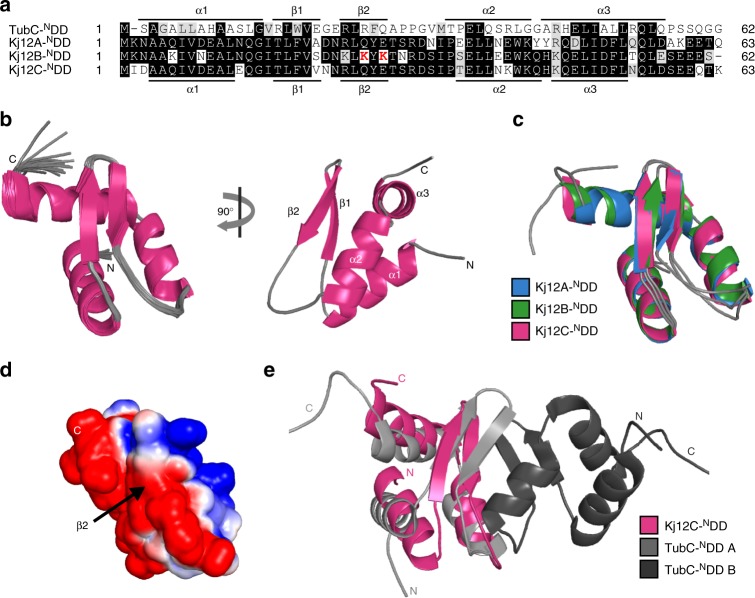
N-terminal docking domains have the same three-dimensional structure. **a** Structure-based sequence alignment of the TubC-^N^DD and the N-terminal docking domains of Kj12ABC. Identical residues are highlighted with dark gray boxes and residues with similar chemical properties are shown in light gray boxes. Key residues for DD interactions are shown in red. The secondary structure based on structural information for TubC-^N^DD and Kj12C-^N^DD is indicated above and below the sequence. **b** Solution structure bundle of the 19 lowest energy conformers and the regularized mean structure for Kj12C-^N^DD. **c** Overlay of cartoon representations of the energy minimized mean structures of Kj12A-^N^DD (blue), Kj12B-^N^DD (green), and Kj12C-^N^DD (magenta). **d** Electrostatic surface potentials of Kj12C-^N^DD mapped on the solvent-accessible surface in the same orientation as in **b** (left) with negatively charged surface areas colored in red, positively charged areas coloured in blue, and white areas corresponding to hydrophobic surfaces. **e** Overlay of the energy-minimized mean structure of Kj12C-^N^DD with one monomer of the TubC-^N^DD dimer

Structural and thermodynamic information about DD interactions in single-module NRPS systems that use their subunits in a nonlinear fashion has previously not been available. The RXP NRPS system from *X. stockiae* KJ12.1 as a model system^15^ consists of the three proteins Kj12A, Kj12B, and Kj12C (Fig. 1a). Bioinformatic analysis of the DDs in this and related RXP NRPSs revealed that all three proteins contain an ^N^DD, and a distinct ^C^DD was found in Kj12A and Kj12B but not in the termination module Kj12C which catalyzes the reaction of the peptide chain with a terminal amine (Supplementary Fig. 2). The three ^N^DDs are ~65 aas long with >70% sequence identity among them (Fig. 2a and Supplementary Fig. 2) and a predicted mixed α/β-secondary structure. Thus these ^N^DDs are much longer and structurally more complex than the previously identified very short and probably unstructured ^N^DDs in the tyrocidin- and surfactin-producing NRPS systems from *Bacillus*^5,6^. Furthermore, all three ^N^DDs showed low sequence homology (<25% identity) to a structurally characterized ^N^DD from the TubC subunit of the tubulysin-synthesizing PKS (TubC-^N^DD) from *Angiococcus disciformis*^13^ that was shown to be a homodimer as well as to a monomeric ^N^DD of subunit B of the epothilone-synthesizing NRPS–PKS system (EpoB ^N^DD) crystallized in its native context as a covalent fusion with the cyclization domain of EpoB^14^. The two ^C^DDs of the modules Kj12A and Kj12B were predicted to be rather short (~20 aas) and unstructured (Supplementary Fig. 2). We therefore decided to determine the structures of all three ^N^DDs excised from the Kj12ABC RXP NRPS system. According to gel filtration in combination with size exclusion chromatography (SEC)–multi-angle static light scattering, all three ^N^DDs were monomeric in solution in contrast to what was observed for the dimeric TubC-^N^DD (Supplementary Fig. 3). Solution-state nuclear magnetic resonance (NMR) using band-selective excitation short-transient (BEST)-transverse relaxation optimized spectroscopy (TROSY)-based pulse sequences and non-uniform sampling rapidly yielded complete NMR resonance assignments for all three ^N^DDs of Kj12ABC. The backbone chemical shift derived from the secondary structures of all three ^N^DDs revealed the presence of three α-helices and two β-strands in the order of α1–β1–β2–α2–α3 (Fig. 2a). The location of the secondary structure elements along the sequence is very similar between the three ^N^DDs as well as to those of the dimeric TubC-^N^DD and the monomeric EpoB-^N^DD^13,14^. The NMR solution structures of the three ^N^DDs from Kj12ABC were solved at very high resolution (backbone root mean square deviation (RMSD) of 0.1–0.2 Å for ordered residues). A complete list of structural statistics according to the recommendations of the NMR-VTF can be found in Supplementary Table 1^16^. The solution structure ensemble of the 19 lowest energy structures calculated with CYANA and an energy-minimized representative mean structure for Kj12C-^N^DD is shown in Fig. 2b. A comparison of the structural ensembles and the mean structures for all three Kj12-^N^DDs are shown in Supplementary Fig. 4. In all three ^N^DDs, β1 and β2 form an antiparallel β-hairpin. Helices α1 and α2 are packed against each other in an antiparallel fashion. They also pack together against one side of the β-hairpin. Helix α3 is separated only by a very short loop (aa 45) from α2 and a sharp kink is introduced in the protein backbone. Thus, α3 crosses the β-hairpin at a ~90° angle. Despite the very similar three-dimensional (3D) structures (RMSDs range from 0.8 to 0.9) of the three ^N^DDs (Fig. 2c), their electrostatic surface potentials differ significantly, which could have an effect on their binding affinities for the ^C^DDs (Fig. 2d and Supplementary Fig. 4). A significant charge difference is found between Kj12B-^N^DD and the other two ^N^DDs on the solvent-exposed side of strand β2 (aa 24–28) where for instance E28 in Kj12A-^N^DD and Kj12C-^N^DD is replaced by a lysine in Kj12B-^N^DD (Supplementary Fig. 4d). The topology of the three RXP-^N^DDs is already known from the TubC-^N^DD structure of *A. disciformis* (Fig. 2e)^13^, but the relative positioning of the secondary structure elements is different between the dimeric TubC-^N^DD and the RXP NRPS-^N^DDs (Cα RMSD of 4.7 Å). In contrast, the Cα RMSD is only 1.3 Å (Supplementary Fig. 4f) between the Kj12C-^N^DD and the monomeric EpoB-^N^DD.

### Interactions with the ^C^DDs

**Fig. 3:**
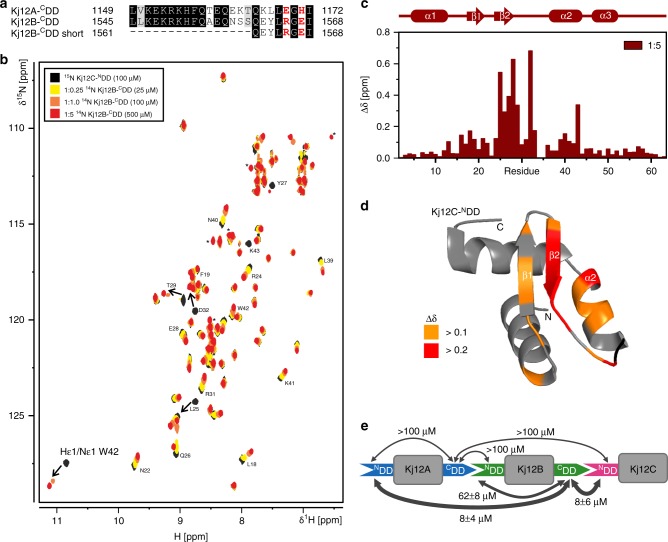
Docking domain interaction. **a** Sequence alignment of Kj12A-^C^DD and Kj12B-^C^DD used in this study. Identical residues are highlighted with dark gray boxes and residues with similar chemical properties are shown in light gray boxes. Key residues for DD interactions are shown in red. **b** Overlay of the ^1^H,^15^N-HSQC spectrum of 100 µM ^15^N-labeled Kj12C-^N^DD in the absence (black) and presence of increasing amounts of unlabeled Kj12B-^C^DD. The molar ratios of the two docking domains are 1:0.25 (yellow), 1:1 (orange), 1:2.5 (dark orange), and 1:5 (red). **c** Histogram of chemical shift changes vs. sequence for Kj12C-^N^DD upon addition of a five-fold molar excess of unlabelled Kj12B-^C^DD with the secondary structure depicted above. **d** Chemical shift changes for Kj12C-^N^DD upon addition of unlabeled Kj12B-^C^DD mapped onto the structure of Kj12C-^N^DD. **e** *K*_d_ values for all DD interactions in the Kj12ABC NRPS as measured by ITC

To further investigate the DD interactions, NMR titration experiments were carried out. All three ^15^N-labeled ^N^DD proteins were titrated with the two unlabeled (^14^N) ^C^DD peptides (Fig. 3a). In Fig. 3b, the titration of Kj12C-^N^DD with Kj12B-^C^DD as followed in ^1^H,^15^N-heteronuclear single quantum correlation (HSQC) experiments is shown as an example. The NMR data for all other titrations are shown in Supplementary Fig. 5. In all six titration experiments, gradual chemical shift changes and/or peak broadening during the stepwise addition of the ^C^DD peptides were observed. This suggests that all three ^N^DDs interacted with both the Kj12A- and the Kj12B-^C^DD, which is in agreement with the observed product spectrum of this NRPS, and form ^N^DD/^C^DD complexes in the fast-to-intermediate exchange regime on the NMR timescale. The chemical shift changes during the titrations were quantified and mapped to the structures of the three ^N^DDs in order to identify the ^C^DD-binding sites. A histogram of chemical shift changes vs. sequence for the titration of the Kj12C-^N^DD with the Kj12B-^C^DD is shown in Fig. 3c. A mapping of these shift changes on the cartoon representation of the Kj12C-^N^DD structure is shown in Fig. 3d (also see Supplementary Figs. 6 and 7 for the data of all six titrations). Unexpectedly, in all six titration experiments, the largest chemical shift changes were always observed for the aas in the β-hairpin and in particular strand β2 and helix α2 of the three ^N^DDs. This suggests that both the Kj12A- and Kj12B-^C^DD bind to all three ^N^DDs at the same sites.

The pairwise interactions between the three ^N^DDs and the two ^C^DDs were further characterized thermodynamically using isothermal titration (ITC) experiments (Supplementary Fig. 8 and Supplementary Table 2). The obtained *K*_d_ values for all interactions (Fig. 3e) are in good agreement with the fast-to-intermediate exchange observed in the NMR titrations. The highest affinity interactions with *K*_d_ values of 8 ± 4 μM and 8 ± 6 µM were observed between the Kj12B-^C^DD and the ^N^DDs of Kj12A and Kj12C, respectively. The Kj12B-^N^DD binds Kj12B-^C^DD with an ~8-fold higher *K*_d_ of 62 ± 8 μM. Kj12A-^C^DD (Fig. 3e) binds only weakly to Kj12C-^N^DD (~100 μM), whereas its interaction with the other two ^N^DDs is too weak to be reliably quantified (Supplementary Fig. 8) The reported affinities for DD pairs in other megasynthases were found to be in a similar range as those observed here^11,13^.

### Solution structure of a DD complex

**Fig. 4:**
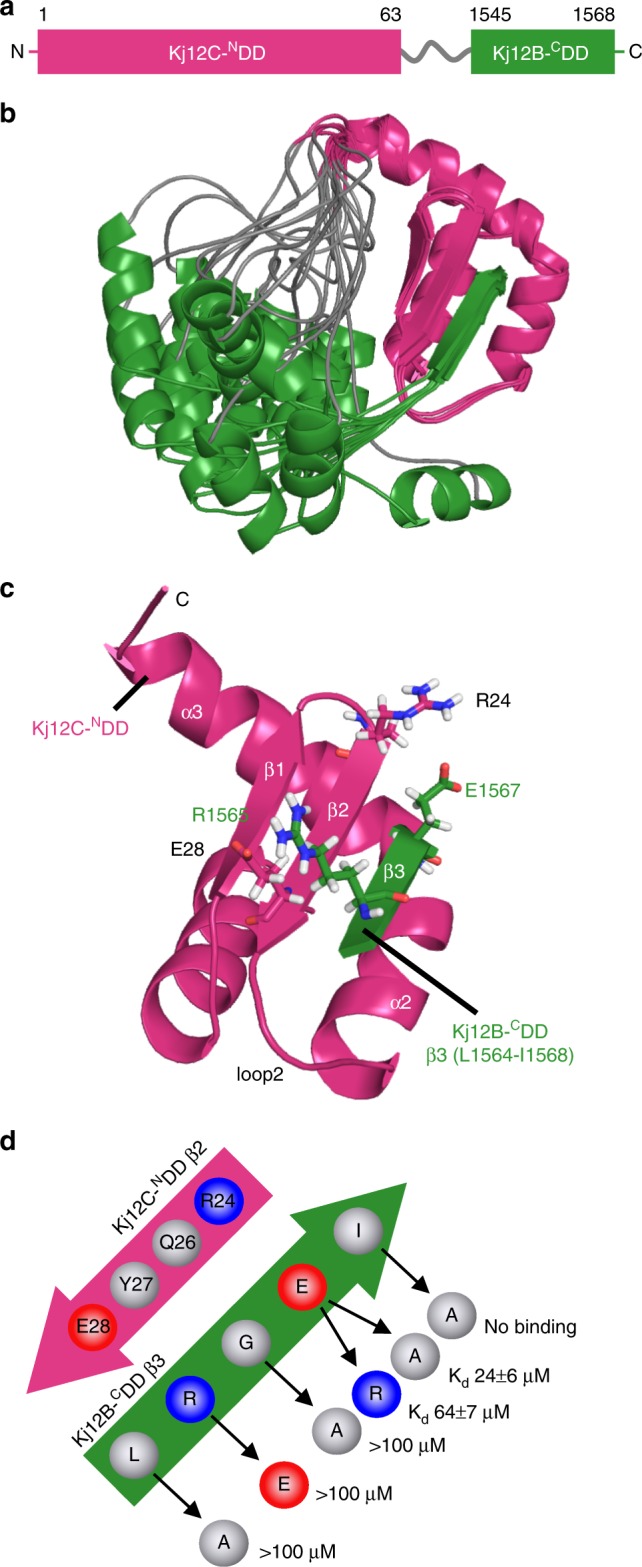
Structure of ^N^DD-^C^DD complex. **a** Schematic representation of the N- and C-terminal docking domain linker construct used in this study with Kj12C-^N^DD in magenta, the 12 amino acid long Gly-Ser linker in gray, and Kj12B-^C^DD (amino acids 1545–1568 from Kj12B corresponding to residues 75–99 in the complex construct Kj12C-^N^DD–12xGS–Kj12B-^C^DD) in green. **b** Solution structure bundle of Kj12C-^N^DD-Kj12B ^C^DD linker construct with colour coding as in **a**. **c** Detailed view of the ^N^DD-^C^DD interaction of β-sheet 2 of Kj12C-^N^DD (magenta) with the last 5 amino acids of Kj12B-^C^DD (green). The charged residues forming salt bridges between the two docking domains are shown in stick representation. **d** Schematic representation of the “recognition rules” for the interaction of β2 of Kj12C-^N^DD (magenta) and β3 of Kj12B-^C^DD (green) in the complex. Positively and negatively charged residues are shown as blue and red circles, respectively, and hydrophobic residues as white circles. *K*_d_ values were determined by ITC titration experiments with synthetic peptides of Kj12B-^C^DD_short_ (Supplementary Table 2 and 4) carrying individual variations of all five residues of β3

Structural information about the interaction of the type of ^N^DD found in the Kj12 RXP NRPS cluster so far is limited to ^C^DD peptide titration experiments for the dimeric TubC-^N^DD. There the interaction surface on the dimeric ^N^DD was identified but no complex structure was obtained^13^. Furthermore, our NMR titration experiments identify a ^C^DD-binding site on the monomeric ^N^DDs that is part of the dimer interface in the TubC-^N^DD. Thus the structural basis for the ^N^DD/^C^DD interactions in the Kj12 RXP NRPS is apparently different from what was observed for TubC. For these reasons and in order to understand the structural basis for the widely different ^N^DD/^C^DD affinities, we solved the structure of Kj12C-^N^DD/Kj12B-^C^DD, the highest affinity DD complex from the Kj12 RXP NRPS. This complex is, however, still in fast-to-intermediate exchange on the NMR timescale and therefore the collection of a large enough number of intermolecular nuclear Overhauser effects (NOEs) is difficult. To overcome this problem, we designed a covalently linked ^N^DD–^C^DD complex with flexible glycine–serine (GS) linkers of different lengths to increase the local ^C^DD concentration at the ^N^DD. To verify that our artificially linked DD pair interacts in *cis* with the same binding mode as the isolated domains in *trans*, we compared the titration end point ^1^H,^15^N-HSQC spectrum of the separate domains with the ^1^H,^15^N-HSQC spectrum of the linked constructs (Supplementary Figs. 9 and 10). For this purpose, we screened different constructs with different linker length (6, 9, and 12 residues) and different domain order (^N^DD-linker- ^C^DD or ^C^DD-linker- ^N^DD). The construct with the longest linker (12 residues) and an ^N^DD–linker-^C^DD arrangement (Fig. 4a) was the best mimic for the natural ^N^DD–^C^DD complex and therefore we solved the structure of this fusion protein (Fig. 4b). To our knowledge, this structure represents the first high-resolution structure of an NRPS DD pair. Surprisingly, the ^N^DD–^C^DD interaction involves only the last five C-terminal aas of the ^C^DD. These aas form an additional β-strand, β3, which interacts in an antiparallel orientation with β2 of the ^N^DD β-sheet as well as with parts of helix α2 (Fig. 4c and Supplementary Fig. 11). The α-helix formed by the first nine aas of the ^C^DD (aa 1543–1555 of Kj12B) does not interact with the ^N^DD (Fig. 4b) and the observed secondary structure is transient, as evidenced by NMR and circular dichroism experiments (Supplementary Fig. 12). An overlay with the structure of the isolated ^N^DD shows that the ^N^DD does not change its conformation in complex with the ^C^DD (Supplementary Fig. 13).

A detailed view of the intermolecular backbone hydrogen bonding interactions between β-strands β2 and β3 from the ^N^DD and the ^C^DD, respectively, is shown in Supplementary Fig. 14. Interestingly, the C-terminal end of the β-sheet of Kj12B ^C^DD (β3) is highly twisted toward helix α2 (Fig. 4c). Thereby, the side chain of the last aa (I1568) is buried in a hydrophobic pocket consisting of the surrounding side chains of helix α2 of the ^N^DD (Supplementary Fig. 11). The interaction of the ^C^DD with this helix explains the large chemical shift changes (Supplementary Fig. 6) observed for aa 39–43 of the ^N^DD during the titration experiments. Additionally, the side chain of the first aa of β3 (L1564) is located in a hydrophobic pocket built by hydrophobic sidechains from β2 and from the loop between β2 and α2 (Supplementary Fig. 11). The signals for the backbone amide groups of the loop residues are either not observable (I34) or have very low intensities in the free ^N^DD (D32, S33) indicative of conformational exchange. In complex with the ^C^DD, these signals have significantly higher intensities. Thus loop2 of the ^N^DD is stabilized upon ^C^DD binding. Y27 of the ^N^DD packs tightly against the small side chain of G1566 in the ^C^DD. The intermolecular interaction between β2 of the ^N^DD and β3 of the ^C^DD is also stabilized by two salt bridges between side chains involving R24 of β2 and E1567 of β3 as well as E28 of β2 and R1565 of β3, respectively (Fig. 4c).

Taken together, ^N^DD/^C^DD complex formation is apparently dependent upon the formation of an intermolecular β-sheet stabilized by two salt bridges and the burial of two large hydrophobic side chains. The involvement of only the five C-terminal aas but not the remainder of the ^C^DD in complex formation is also supported by steady-state {^1^H}-^15^N heteronuclear Overhauser effect (hetNOE) measurements. As expected, low hetNOE values for residues in the GS linker and in the majority of the ^C^DD including the residues of the transient a-helix formed by aa 1543–1555 demonstrate that these aa residues are highly flexible in solution. Only the C-terminal aas of the ^C^DD that are part of β3 have the same high hetNOE values as the conformationally rigid residues of the ^N^DD (Supplementary Fig. 12). To further verify that the ^N^DD–^C^DD interaction is exclusively based on this β-sheet interaction, we repeated the titration experiments with a shortened Kj12B-^C^DD peptide that comprises only the β-sheet (eight C-terminal residues from Kj12B-^C^DD, aa 1561–1568, Fig. 3a). The end points of both NMR titration experiments (Kj12C-^N^DD with Kj12B-^C^DD and Kj12B-^C^DD_short_, respectively, in identical concentrations and ratios) overlapped perfectly (Supplementary Fig. 15) and ITC measurements confirmed that the short ^C^DD peptide binds with a very similar affinity (15 ± 3 μM) compared to the original longer ^C^DD peptide (Supplementary Fig. 15).

In order to test the relative importance of the observed intermolecular interactions, variants of the Kj12B-^C^DD_short_ peptide were synthesized (Figure 4d, Supplementary Table 4, Supplementary Fig. 16) and tested. Replacement of either of the two large hydrophobic side chains L1564 and I1567 by the smaller alanine led to a significant loss of affinity (Supplementary Fig. 17 and Supplementary Table 2). Increasing the size of the side chain of G1565 that stacks against Y27 by replacement with alanine also leads to a decrease in binding affinity. Importantly, breaking of the salt bridges between E28 and R1565 and between R24 and E1567, respectively, significantly lowers the affinities between the Kj12C-^N^DD and the ^C^DD peptide. In particular, the salt bridge between E28 and R1565 seems to contribute strongly to the binding affinity.

**Fig. 5:**
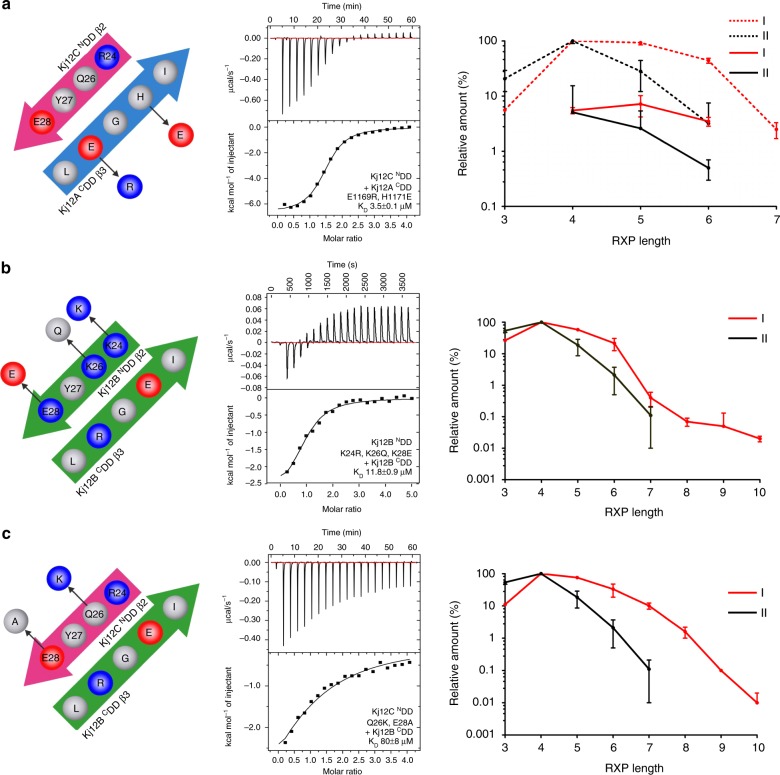
Optimization of ^C^DD or ^N^DDs in Kj12ABC system for the production of longer RXPs. Additionally, the ITC thermograms and the derived binding curves for titrations between optimized ^C^DD or ^N^DDs variants are shown. **a** Kj12A-^C^DD was optimized by two amino acid exchanges, E1169R and H1171E, on Kj12A-^C^DD. Co-expression of natural Kj12B with optimized Kj12C led to increased production of longer RXPs. Red lines (I) represent RXP production in the modified system, black lines (II) represent RXP production in the natural Kj12BC. Solid lines indicate fully methylated Val (mV) RXPs, dashed lines indicate RXPs containing only one non-methylated Val. **b** Optimization of Kj12B-^N^DD for tighter interaction with Kj12B-^C^DD via three amino acid exchanges, K26Q, K24R and K28E, on Kj12B-^N^DD. A red line (I) represents RXP production in the optimized system, a black line (II) represents RXP production in the natural Kj12BC. Only fully methylated RXPs are shown. **c** In addition to amino acid exchanges in **b**, Kj12C-^N^DD was additionally modified via two amino acid exchanges, Q26K and E28A, to reduce the interaction with Kj12B-^C^DD and allowing better interaction between Kj12B-^N^DD and Kj12B-^C^DD. No RXPs were detected after co-expression of natural Kj12B and modified Kj12C-^N^DD probably due to very weak affinities. A red line (I) represents RXP production in the optimized system, a black line (II) represents RXP production in natural Kj12BC. Only fully methylated RXPs are shown. *x* Axis, numbers of amino acid residues in RXPs (RXP length). *y* Axis, production of the corresponding RXPs relative to the most abundant derivative set to 100%

In this respect, it is interesting to note that in the Kj12B-^N^DD the equivalent of E28 is replaced by lysine (Fig. 2a) explaining the lower affinity measured for its interaction with the native Kj12B-^C^DD peptide. In contrast, a Kj12B-^C^DD peptide R1565E that should restore salt bridge formation with K28 in Kj12B-^N^DD binds with a much higher affinity to the Kj12B-^N^DD (13 ± 1 μM) (Supplementary Table 2). Furthermore, these data rationalize why the Kj12A-^C^DD binds rather weakly to all three ^N^DDs. At the positions corresponding to E1567 and R1565 in the Kj12B-^C^DD, the Kj12A-^C^DD contains a histidine and a glutamate residue, respectively (Fig. 3a). Thus the formation of the two intermolecular salt bridges across the intermolecular β-sheet is weakened or prevented in the interactions involving the Kj12A-^C^DD. In contrast, a Kj12A-^C^DD peptide with an E1169R and a H1171E double mutant supporting the formation of both salt bridges binds with high affinity (3.5 ± 0.1 μM) to the Kj12C-^N^DD (Fig. 5a, Supplementary Table 2).

We also tested whether the ^N^DD–^C^DD interaction is influenced by adjacent domains in the respective modules. To this end, we recombinantly produced the Kj12B-^C^DD fused to its preceding T domain (Kj12B-Tdom-^C^DD) and the full-length Kj12C module consisting of the ^N^DD and the entire condensation domain (Kj12C-^N^DD-C_term_) (Supplementary Fig. 18). ITC experiments showed that the presence of the Kj12B T domain does not influence the affinity of the Kj12B ^C^DD for the Kj12C ^N^DD and that the full-length Kj12C module binds with the same affinity to the Kj12B ^C^DD_short_ peptide as the isolated Kj12C ^N^DD (Supplementary Fig. 18 and Supplementary Table 2). The Kj12C module without the ^N^DD shows no binding to the Kj12B ^C^DD_short_ peptide. Thus our results suggest that the tuning of intermolecular DD interactions by modulating the formation of the two intermolecular salt bridges in the context of full-length modules could influence the interactions between entire NRPS modules and thereby the frequency of intermodular substrate transfer and the resulting product spectrum in this NRPS system.

### DD reprogramming results in peptide diversification in vivo

In order to verify the functional importance of the identified key residues for DD interactions in the context of the entire Kj12ABC system in vivo, we systematically changed selected positions in the DDs and analyzed the RXP profile in *E. coli* expressing the complete Kj12ABC NRPS system with either the parent or the modified DDs. As a starting point, in Kj12A the ^C^DD was changed to E1169R and H1171E in order to increase the affinity between Kj12A-^C^DD and Kj12C-^N^DD (Fig. 5a) in analogy to the in vitro peptide-binding experiments described above (Supplementary Fig. 19d, Supplementary Table 2). Indeed, the optimized Kj12A variant was able to interact with Kj12C resulting in the formation of RXP **1**, V-PEA not detected in the native Kj12AC system (Supplementary Fig. 20). When the optimized Kj12A was combined with native Kj12BC, an increase of longer RXPs was observed (Fig. 5a and Supplementary Fig. 21) that was also observed for an artificial system where the aa specificity of Kj12B was changed from V to L allowing an easier differentiation of the activities of Kj12A and Kj12B (Supplementary Fig. 22). Since it was previously shown that peptide length depends on the protein stoichiometry between Kj12B (elongation) and Kj12C (termination)^13^, this likely results from more efficient binding between Kj12A and Kj12C. Thus, less Kj12C is available for peptide termination via binding to Kj12B.

A similar production of longer peptides with a chain length of up to ten aas was observed in a Kj12BC system when the ^N^DD of Kj12B- was optimized via K28E, K26Q, and K24R exchange for a higher affinity toward Kj12B-^C^DD (Fig. 5b and Supplementary Fig. 23). The higher affinity of these Kj12B-^N^DD mutants toward the Kj12B-^C^DD was confirmed in vitro in ITC measurements (Supplementary Fig. 19). Aa exchange of E28A and Q26K in the ^N^DD of Kj12C designed to weaken the interaction between the Kj12B-^C^DD and the Kj12C-^N^DD and thereby to reduce termination also resulted in longer peptides of up to nine aas in a Kj12BC system (Supplementary Fig. 24). When the optimized sequences for Kj12B-^N^DD (K28E, K26Q, and K28E) and Kj12C-^N^DD (E28A and Q26K) were combined, even more long-chain peptides were produced (Fig. 5c and Supplementary Fig. 24).

### Prediction of DD interactions in other megasynthase systems

In order to investigate whether the DD recognition mode of the RXP-NRPS system observed here also occurs in other systems, a BLASTP search using Kj12C-^N^DD and TubC-^N^DD as query sequences was carried out. In *Xenorhabdus* strains, besides highly related RXP-NRPS systems, a new family of NRPS related to the taxlllaid lipopeptide-producing NRPS TxlAB^17^ was identified. Here DDs connecting NRPS subunits carrying C-terminal epimerization (E) to N-terminal condensation (C) domains (Supplementary Fig. 2) apparently interact in a fashion similar to that observed in Kj12ABC.

Moreover, DD pairs with a very similar putative interaction mode were identified in *Pseudomonas*, *Janthinobacterium*, *Paenibacillus*, *Acidobacterium*, *Mycolicibacterium*, cyanobacteria, and myxobacteria (Supplementary Fig. 25, Supplementary Table 3).

In different myxobacteria and cyanobacteria, the ^C^DD–^N^DD pair was found to connect NRPS or PKS (…-T-^C^DD) with NRPS (^N^DD-C-…) in different PKS/NRPS and NRPS systems (e.g., melithiazol^18^, tubulysin, micropeptin^19^, and cyanopeptolin^20^). In *Acidobacterium*, the DD pair was found as part of a large PKS/NRPS system where it connects a PKS with an NRPS subunit and in the Gram-positive *Mycolicibacterium* where it connects a stand-alone thioesterase (TE) domain with an NRPS. In *Pseudomonas*, it is part of a conserved but not further characterized PKS/NRPS system of which the produced natural product is not known yet. There it connects an NRPS carrying a C-terminal oxidoreductase domain with an NRPS carrying an N-terminal C domain (Supplementary Table 3). A DD pair highly similar to that in Kj12ABC is also present in the PKS/NRPS system responsible for romidepsin (FK228, Istodax®) production in *Chromobacterium violaceum*, where it connects the PKS (DepC) with the NRPS (DepD) part^21^. Romidepsin is a known histone deacetylase inhibitor used as anticancer agent in cutaneous and peripheral T cell lymphomas^22^.

In all these systems, putative interactions between ^N^DDs and ^C^DDs dependent on salt bridges and hydrophobic interactions similar to the Kj12ABC system could be predicted (Supplementary Fig. 25). However, in some examples as in *Janthinobacterium* (uncharacterized NRPS system), the polarity of the salt bridges is reversed. In other cases, a salt bridge is replaced by a hydrogen bond or by a hydrophobic interaction.

## Discussion

Engineering of NRPS or PKS systems for the production of derivatives or even new natural products requires not only efficient modification of catalytic domains^23,24^ but also modification of protein–protein interactions within megasynthases that comprise multiple subunits, as is the case for most systems that incorporate >5 building blocks.

Here we have analyzed the DD-mediated interactions required for the production of rhabdopeptides using RXP-type NRPS subunits from *Xenorhabdus* KJ12.1 as a model system. We demonstrated that aa exchanges in the respective DDs not only results in a predicted shift in protein affinity in vitro but also in the production of different peptides in vivo.

From an overlay of the TubC-^N^DD structure with that of Kj12C-^N^DD, it is obvious that the ^C^DD-binding sites must be very different in the two systems (Supplementary Fig. 4e). This is due to the dimeric structure of the TubC-^N^DD where the β-hairpin forms part of the dimerization surface (Fig. 2e) leaving no space for interaction with a ^C^DD as observed in Kj12ABC. However, from a brief analysis of different biosynthetic gene clusters it was obvious that several other megasynthases apparently use ^N^DD/^C^DD interactions that are structurally similar to what we found for Kj12ABC (Supplementary Figs. 2 and 25) and conform to similar recognition rules. The identified DD pairs link different NRPS and PKS enzymes in several different bacterial genera and taxa and thus might be a useful tool for megasynthase engineering in the future.

An understanding of the structural principles governing megasynthase DD interactions forms a basis for future engineering approaches of such systems since it might allow the combination of megasynthase subunits from different biosynthetic pathways that have been individually optimized in a combinatorial approach. Also the splitting of large megasynthase modules that are too difficult to engineer as intact units into smaller functional units might be possible using well-studied DDs. Additionally, DD-engineered crosstalk of megasynthase subunits from different biosynthesis pathways could even increase the chemical diversity of currently known natural products, while the DD specificity code described here allows for fast identification of specific protein–protein interactions and thus might help to elucidate biosynthesis pathways for systems that are not collinear.

However, it should be mentioned that the DDs described in this work and also found in the taxlllaid-producing NRPS can link E and C domains (…-E-^C^DD to ^N^DD-C-…) as it is also the case for the DDs described in the tyrocidine- and surfactin-producing NRPS in *Bacillus*^5,6^. However, despite their functional similarity the structural basis for their interaction in *Bacillus* and *Xenorhabdus* is very different due to differences in DD length, folding, and aa composition suggesting that these interaction domains might indeed be orthogonal and thus represent interesting tools for synthetic biology of NRPS and beyond. From our brief bioinformatics analysis of DDs in other NRPS clusters, it is evident that, while the RXP type of DD interactions is also found in other systems, additional DD types exist for NRPS and other assembly lines that require further structural and biochemical analysis enabling their future use in NRPS engineering or understanding the basic principles of these megasynthase pathways.

## Methods

### General molecular biology

Cultivation of *Xenorhabdus* and *Escherichia coli* strains (Supplementary Table 5) was performed in LB medium following standard protocols^15^. Procedures, such as plasmid DNA preparation, transformation, restriction digestion, and DNA gel electrophoresis, were adapted from standard protocols^25^. Isolation of genomic DNA was carried out according to the manufacturer’s instructions (QIAGEN). Phusion High-Fidelity DNA-Polymerase (Thermo Scientific) was used for PCR amplifications. PCR primers used in this study are listed in Supplementary Table 6. All the plasmids (Supplementary Table 7) generated in this study were constructed via Gibson assembly^26^. The basic cloning was performed in *E. coli* DH10B MtaA.

### Expression and purification of DDs and DD complexes

For structure determination, ^N^DDs from *X. stockiae* Kj12ABC as well as the covalently linked ^N^DD–^C^DD complexes and ^N^DD mutants were heterologously expressed in *E. coli* BL21-Gold(DE3) under the control of a T7 promoter. The coding sequences were cloned into a modified pET11a vector containing an N-terminal His_6_-SUMO tag. DNA fragments encoding Kj12C-^N^DD and Kj12B-^C^DD were linked with a 12 aa long GS linker in between. The resulting constructs were grown in uniformly ^15^N and ^15^N,^13^C M9 minimal media containing 1 g L^−1^ ^15^NH_4_Cl (Cambridge Isotope Laboratories) or 1 g L^−1^ ^15^NH_4_Cl and 2.5 g L^−1^ ^13^C_6_-d-glucose (Cambridge Isotope Laboratories) and 100 mg mL^−1^ ampicillin. For ITC measurements, proteins were expressed in *E. coli* BL21-Gold (DE3) using LB medium. Protein expression was induced at an OD_600_ of 0.8 with 1 mM IPTG overnight at 25 °C. After expression, cells were lysed by sonication in lysis buffer containing 50 mM Tris–HCl, pH 8.0, 300 mM NaCl, one spatula tip of Dnase, RNase (Roche), and protease inhibitor (Roche). The lysate was cleared by centrifugation (30 min, 8000 × *g*, 4 °C) and the supernatant was passed through a HisTrap HP column (GE Healthcare) using HisTrap-buffer (50 mM sodium phosphate buffer at pH 8 with 30 mM NaCl and 500 mM imidazole for elution). The His_6_-SUMO tag was cleaved off by Ulp1 protease treatment in dialysis buffer (50 mM Tris/HCl, pH 8, 50 mM NaCl) and removed by a second purification step with the HisTrap HP column using HisTrap-buffer. All three ^N^DDs and the ^N^DD-^C^DD complexes were further purified via ion exchange chromatography (IEX) with a Q-Sepharose anion exchange column (HiPrep 16/10 column, GE Healthcare, IEX binding buffer: 50 mM Tris/HCl, pH 8, 50 mM NaCl, IEX elution buffer 50 mM Tris/HCl, pH 8, 1 M NaCl) followed by SEC on a HiPrep 16/60 Sephacryl S-100 High Resolution column (GE Healthcare) in SEC buffer and 50 mM sodium phosphate buffer at pH 6.5 with 100 mM NaCl.

The coding sequences for Kj12C-^N^DD with the terminal condensation domain (Kj12C-^N^DD-Cterm) and a construct lacking the ^N^DD (Kj12C-Cterm) were cloned into a pCOLA vector with a kanamycin resistance gene and a C-terminal His_6_-tag with a TEV cleavage site. The coding sequence for the thiolation domain of Kj12B together with the ^C^DD (Kj12B-Tdom-^C^DD, aa 1459–1568) was cloned into a modified pET11a vector containing an N-terminal His_6_-SUMO tag. Constructs were heterologously expressed in *E. coli* BL21-Gold(DE3) using LB medium for cultivation. Protein expression was induced at an OD_600_ of 0.8 with 1 mM IPTG overnight at 20 °C. After expression, cells were lysed by sonication in lysis buffer containing 50 mM Tris–HCl, pH 8.0, 300 mM NaCl, one spatula tip of DNase, RNase (Roche), and protease inhibitor (Roche). The lysate was cleared by centrifugation (30 min, 8000 × *g*, 4 °C) and the supernatant was passed through a HisTrap HP column (GE Healthcare). Kj12B-Tdom-^C^DD was further purified via Q-Sepharose anion exchange (HiPrep 16/10 column, GE Healthcare) using IEX buffers followed by SEC on a HiPrep 16/60 Sephacryl S-100 High Resolution column (GE Healthcare) using 50 mM Tris/HCl, pH 8 with 100 mM NaCl. The His_6_-tag was cleaved off by TEV protease treatment in dialysis buffer and removed by a second purification step with the HisTrap HP column. SEC for Kj12C-^N^DD-Cterm and Kj12C-Cterm constructs was performed on a HiPrep 16/60 Sephacryl S-200 High Resolution column (GE Healthcare) using SEC buffer with 2 mM β-Mercaptoethanol.

### Construction of plasmids encoding modified NRPS subunits

All constructs for the production of modules containing DDs with exchanged aas (1–3 exchanges) were designed using oligonucleotides carrying the required mutations. For example, for the production of the Kj12A-^C^DD carrying E1169R and H1171E, Gibson assembly of the required PCR fragments into the vector pCOLA-ara-tacI was used and similar approaches were used also for the production of the plasmids encoding Kj12B modules with an -^N^DD with single (K28E or K26Q), double (K28E and K24R or K26Q and K28E) and triple (K28E, K24R, and K26Q) aa exchanges, as well as Kj12C-^N^DD with single (E28K or E28A) or double (Q26K and E28A) aa exchanges to weaken the interaction with other ^C^DDs.

### Heterologous production of RXPs in *E. coli* DH10B MtaA

To compare the metabolic profiles after modifications of ^N^DDs and/or ^C^DDs, *E. coli* DH10B MtaA strain was separately transformed with a mixture of different plasmid pairs carrying full-length modules with optimized Kj12A-^C^DD, Kj12B-^N^DD, modified Kj12C-^N^DD, or those carrying the original DDs. The resulting strains were individually inoculated into 5 mL of liquid LB media supplemented with appropriate antibiotics (kanamycin, 50 μg/mL; chloramphenicol, 34 μg/mL and spectinomycin, 50 μg/mL) and cultivated at 30 °C overnight with shaking at 200 rpm. The next day, 100 μL of overnight culture was transferred into 10 mL fresh LB medium with corresponding antibiotics, 1 mM PEA, 2% (v/v) of Amberlite XAD-16 resin (Sigma-Aldrich), 0.1% of l-arabinose for inducing the expression of RXP-NRPSs, and followed by growing the culture at 30 °C, 1 day, 200 rpm for production of RXPs.

### Culture extraction

The bacterial cell pellets and XAD beads were collected after centrifugation and resuspended in 10 mL of methanol. XAD beads were washed with methanol by inverting for 1 h and followed by separating from methanol through filter paper. The resulting methanol extracts were evaporated to dryness and dissolved in 1 mL of methanol.

### High-performance liquid chromatography–mass spectrometry (HPLC-MS) analysis

The methanol extracts obtained above (1 mL) were centrifuged at 17,000 × *g* for 20 min. Twenty μL of crude extracts were diluted in 180 μL methanol before analysis, 5 μL of which was injected and analyzed by electrospray ionization–HPLC-MS by a Dionex UltiMate 3000 system coupled to a Bruker AmaZon X mass spectrometer with an ACQUITY UPLC™ BEH C18 column (130 Å, 2.1 × 100 mm^2^, 1.7 µm particle size, Waters GmbH) at a flow rate of 0.6 mL min^−1^ using acetonitrile (ACN) and water containing 0.1% formic acid (v/v) in a gradient ranging from 5 to 95% of ACN over 16 min. BPC spectra for RXPs were recorded in positive ion mode with the range from 80 to 1600 *m/z* and ultraviolet (UV) at 200–600 nm.

### Chemical synthesis of short peptides

For a schematic overview, see Supplementary Fig. 16 showing the synthesis of QEYARGEI as an example. Step **a** was loading of the first aa (Ile) on the 2-chlorotrityl chloride (2-CTC) resin. A solution of Fmoc-Ile-OH (212 mg, 0.6 mmol, 3 eq.) and *N,N*-diisopropylethylamine (DIPEA, 0.31 mL, 1.8 mmol, 9 eq.) in 4 mL dry dichloromethane (DCM) was placed in a plastic reactor vessel filled with 2-CTC resin (125 mg, 0.2 mmol, 1.0 eq.). The resulting mixture was incubated at room temperature overnight. The remaining free binding sites were capped upon incubating twice with a mixture of 80% DCM, 15% CH_3_OH, and 5% DIPEA for 10 min at room temperature. The resin was washed several times with dimethylformamide (DMF), CH_3_OH, and DCM and treated with 20% piperidine in *N*-methylpyrrolidone (NMP, 3 × 10 min) to remove the Fmoc-protecting group. The combined filtrates were used to determine the actual loading of the resin at *λ*_301 nm_. Afterwards, the resin was washed with DCM and dried. Step **b** is solid-phase peptide synthesis. The linear sequence was synthesized on the preloaded Ile-2-CTC resin on a 25-µmol scale with a Syro Wave peptide synthesizer by using standard Fmoc/*t*-Bu chemistry. The resin was placed in a plastic reactor vessel with a Teflon frit and an amount of 6 eq. of aa derivatives (Fmoc-Glu(OtBu)-OH, Fmoc-Gly-OH, Fmoc-Arg(Pbf)-OH, Fmoc-Ala-OH, Fmoc-Tyr(tBu)-OH, Fmoc-Gln(Trt)-OH, 0.2 M) was activated in situ at room temperature with 6 eq. of *O*-(6-chlorobenzotriazol-1-yl)-*N,N,N*’*,N*’-tetramethyluronium hexafluorophosphate (0.6 M) in DMF in the presence of 12 eq. DIPEA (2.4 M) in NMP for 50 min. Fmoc-protecting groups were removed with a solution of 40% piperidine in NMP for 5 min and the deprotection step was repeated for another 10 min with 20% piperidine in NMP. After each coupling and deprotection step, the resin was washed with NMP. After the addition of the final residue, the resin was washed with NMP, DMF, and DCM and dried. Step **c** is cleavage of peptide from the resin. A total of 1 mL 95% trifluoroacetic acid and 2.5% triisopropylsilane in water was added to the peptidyl resin (25 µmol) and the mixture was agitated for at least 3 h at room temperature. The resin was removed by filtration and the crude peptide was precipitated in cold diethyl ether/petrolether (2:1). After centrifugation (4000 rpm, 10 min, 4 °C) and careful decantation of the supernatant, the crude peptide was washed with diethyl ether twice, centrifuged, and dried. The peptide was further purified by preparative HPLC system and confirmed by HPLC/MS and high-resolution MS (QEYARGEI, mass calc. 483.2380 [M + 2 H]^2+^, found 483.2382 [M + 2 H]^2+^).

### NMR spectroscopy

For NMR measurements, the DDs (0.5–1 mM) were prepared in 50 mM sodium phosphate buffer pH 6.5, 100 mM NaCl, and 10% D_2_O. For NMR titration experiments, a protein concentration of 100 µM was used. NMR spectra were acquired at 20 °C on Bruker AVANCE III 600, 700, 800, and 950 MHz spectrometers equipped with cryogenic triple resonance probes. The proton chemical shifts were internally referenced to 2,2-dimethyl-2-silapentane-5-sulfonic acid and the heteronuclear ^13^C and ^15^N chemical shifts were indirectly referenced with the appropriate conversion factors^27^. The standard set of triple resonance experiments (HNCO, HN(CO)CA, HNCACB) was used for the backbone resonance assignments of the ^N^DD of Kj12C^28^. For the other ^N^DDs and the ^N^DD-^C^DD linker constructs, BEST-TROSY versions of the triple resonance spectra were used together with non-uniform sampling^29^. Shaped proton pulses with a bandwidth of 5.0 ppm centered at 8.5 ppm were used. The delay between scans was set to 0.3 s in all experiments. Non-uniform sampling was employed in all BEST-TROSY 3D experiments, where the percentage of data points of the full *t*_1_/*t*_2_ grid actually acquired varied between 25 and 30 with T_2_ weighting of 0.05 s in the ^15^N dimension and 0.01 s in the ^13^C dimension, respectively. Processing of 3D data sets with non-uniform sampling was carried out with the multidimensional decomposition (mdd) algorithm provided by TopSpin 3.5 using default parameters. For side chain resonance assignment, 3D HBHA(CO)NH, (H)CCH-TOCSY, and H(C)CH-TOCSY experiments were used. All spectra were recorded and processed using Bruker TopSpinTM 3.5 and analyzed using the programs CARA^30^ and CcpNmr Analysis^31^. {^1^H}-^15^N-hetNOE experiments^32^ were carried out for the ^15^N-labeled ^N^DD of Kj12C and the ^15^N-labeled ^N^DD-^C^DD linked complex construct and recorded using standard Bruker pulse sequences. Experiments were run twice in an interleaved fashion with and without proton saturation during the recovery delay. Peak intensity differences were obtained using Bruker TopSpinTM 3.5 for peak integration and the peak intensity ratio was calculated as *I* = *I*_X_/*I*_0_. For titration experiments with NMR, ^C^DD peptides (Kj12A-^C^DD, YLVKEKRKHFQTEQEKTQKLLFGHI; and Kj12B-^C^DD, LLKEKRKHFQAEQNSSQEYLRGEI), both from JPT Peptides Technologies GmbH, or the short peptide variants were lyophilized twice and dissolved in water to measure the peptide concentrations with UV-vis spectroscopy. Therefore, the Kj12A-^C^DD peptide contains a non-native N-terminal tyrosine. For titration experiments, ^1^H,^15^N HSQCs or ^1^H,^15^N BEST-TROSY-HSQCs were recorded after each successive addition of unlabeled ^C^DD (25–500 µM) to a 100 µM ^15^N ^N^DD protein sample in the identical buffer. To evaluate NMR titration experiments, the chemical shifts were determined using the peak picking function of CcpNmr Analysis^31^. The chemical shift differences were calculated using the following function^33^:

1 $$\Delta \delta = \sqrt {\Delta \delta _{\mathrm{H}}^2 + \left( {\frac{{\Delta \delta _{\mathrm{N}}}}{{6.5}}} \right)^2}.$$

### Structure calculation

^15^N-nuclear Overhauser spectroscopy (NOESY)-HSQC, ^13^C-NOESY-HSQC (aliphatic carbons), and ^13^C-NOESY- HSQC (aromatic carbons) experiments in H_2_O with mixing times of 250 ms were used to obtain distance restraints. The TALOS-N server was used to generate torsion angle restraints^34^ based on backbone H, N, Cα, Cβ, and CO chemical shifts and used for all residues with ^15^N hetNOE values >0.5. Peak picking and NOE assignment was performed with the ATNOS/CANDID module in UNIO^35^ in combination with CYANA^36^ using the 3D NOESY spectra. To correct falsely picked artifacts, the peak lists were reviewed manually and corrected. Distance restrains were obtained using the automated NOE assignment and structure calculation protocol available in CYANA^36^. An assignment of >93% of the observable NOESY crosspeaks for all NOESY spectra was achieved. Restrained energy refinement with OPALp^37^ and the AMBER94 force field^38^ of the 20 structures with the lowest target function was carried out. With the CYANA “regularized”-macro, a single representative structure was obtained^39^. This representative mean structure as well as the 19 conformers with the lowest CYANA target function were used for the structure validation with the Protein Structure Validation Software suite1.5^40^ restricted to residues with hetNOE values >0.6. Electrostatic surface potential calculations were conducted with the PDB2PQR web server^41^ using the PARSE force field and visualized with the APBS plug-in^42^ for PyMOL with a threshold for electrostatic potential shading from −1 k*T*/*e* to +1 kT/*e* (k = Boltzmann’s constant, T = absolute temperature, and e = electron charge (The PyMOL Molecular Graphics System, Version 2.0 Schrödinger, LLC). All figures of structures were prepared with PyMOL.

### ITC calorimetry

ITC measurements were performed at 20 °C in 50 mM sodium phosphate buffer, pH 6.5, and 100 mM NaCl using a MicroCal iTC200 (Malvern Instruments) or a Nano ITC Low Volume (TA Instruments) calorimeter. In all, 50 µM of the ^N^DDs were titrated with 1 mM of the ^C^DD peptides and their variants. In order to measure the affinity of the Kj12B T-domain-^C^DD for the Kj12C ^N^DD, a 50 mM solution of the Kj12B T-domain-^C^DD construct was placed in the cell and titrated with a 1 mM solution of the Kj12C ^N^DD in the syringe due to the lower solubility of the Kj12B T-domain-^C^DD construct. NMR experiments showed that Kj12C ^N^DD does not aggregate at a concentration of 1 mM and remains well folded. ITC experiments started with an initial waiting time of 120 s. The first injection of 0.2 µl was followed by 19 serial injections of 2 µl, separated by an interval of 180 s. For each experiment, the reference power was set to 11 µcal^−1^, stirring speed to 750 rpm (75 rpm for the Nano ITC), and the high feedback mode was selected. Three independent titrations were performed for each combination of DDs. The thermograms were processed using Origin7.0 (OriginLab) or NanoAnalyze Data Analysis 3.7.5 (TA Instruments) assuming a one site binding model. For the all ITC measurements where the protein was saturated with peptide during the final titration steps and a clear plateau was reached in the titrations, this plateau was used for baseline correction. In titrations of the Kj12B-^N^DD with Kj12B-^C^DD and Kj12B-^C^DD E1567R, respectively, full saturation was not reached. In these cases, the ^C^DD peptides were titrated into buffer and the resulting thermograms were subtracted from those of the actual titration. *c*-Values were calculated by using Eq. (2):

2 $$c = n{\mathrm{K}}_{\mathrm{a}}[{\mathrm{M}}]_{\mathrm{T}}$$

where K_a_ is the binding constant, [M]_T_ the total macromolecular concentration in the cell, and *n* is the stoichiometry of interaction^43^. Reliable binding constants were determined for ITC data with *c*-values >1 and are listed in Supplementary Table 2. For ITC data with *c*-values <1, the determination of binding constants is not reliable^44^. If there is apparent binding in the ITC curves and in NMR titration experiments, we labeled these interactions only qualitatively as weak binding.

### Alignments

Sequence alignments of ^C^DDs were carried out with the multiple sequence alignment tool Clustal Omega^45^. Owing to the low sequence identity between RXP ^N^DDs and the tubulysin DD (TubC-^N^DD), a structural alignment was done with the Espresso mode of the T-Coffee alignment web server^46^. All alignments were visualized with Boxshade.

### CD measurements

CD measurements were carried out on a Jasco-815 CD spectrometer (Jasco, Gross-Umstadt, Germany) with 1 mm quartz cuvettes. In all, 50 µM peptide samples were dissolved in 10 mM sodium phosphate buffer at pH 6.5 with 25 mM sodium chloride. Baseline corrections were performed automatically and automatic averaging of three measurements was performed. Spectra were recorded at 293 K in a spectral range between 190 and 290 nm with 1 nm scanning intervals, 5 nm bandwidth and 50 nm min^−1^ scanning speed.

## Electronic supplementary material

Supplementary Information

## Data Availability

All structures have been deposited in the Protein Data Bank under the ID codes 6EWS (Kj12A-^N^DD), 6EWT (Kj12B-^N^DD), 6EWU (Kj12C-^N^DD), and 6EWV (Kj12- ^N^DD-Kj12B-^C^DD). Other data are available from the corresponding authors upon reasonable request.
