# Supplementary material for: Structure-based redesign of docking domain interactions modulates the product spectrum of a rhabdopeptide-synthesizing NRPS
Source: Nat Commun. 2018 Oct 19;9:4366. doi: 10.1038/s41467-018-06712-1 (PMC6195595; doi:10.1038/s41467-018-06712-1)
Supplement: Supplementary file 1 — Supplementary Information [file 41467_2018_6712_MOESM1_ESM.pdf]

Supporting information

Structure-based redesign of docking domain interactions modulates the product spectrum of a rhabdopeptide-synthesizing NRPS

Carolin Hacker, Xiaofeng Cai *et al*.

Supplementary Figures

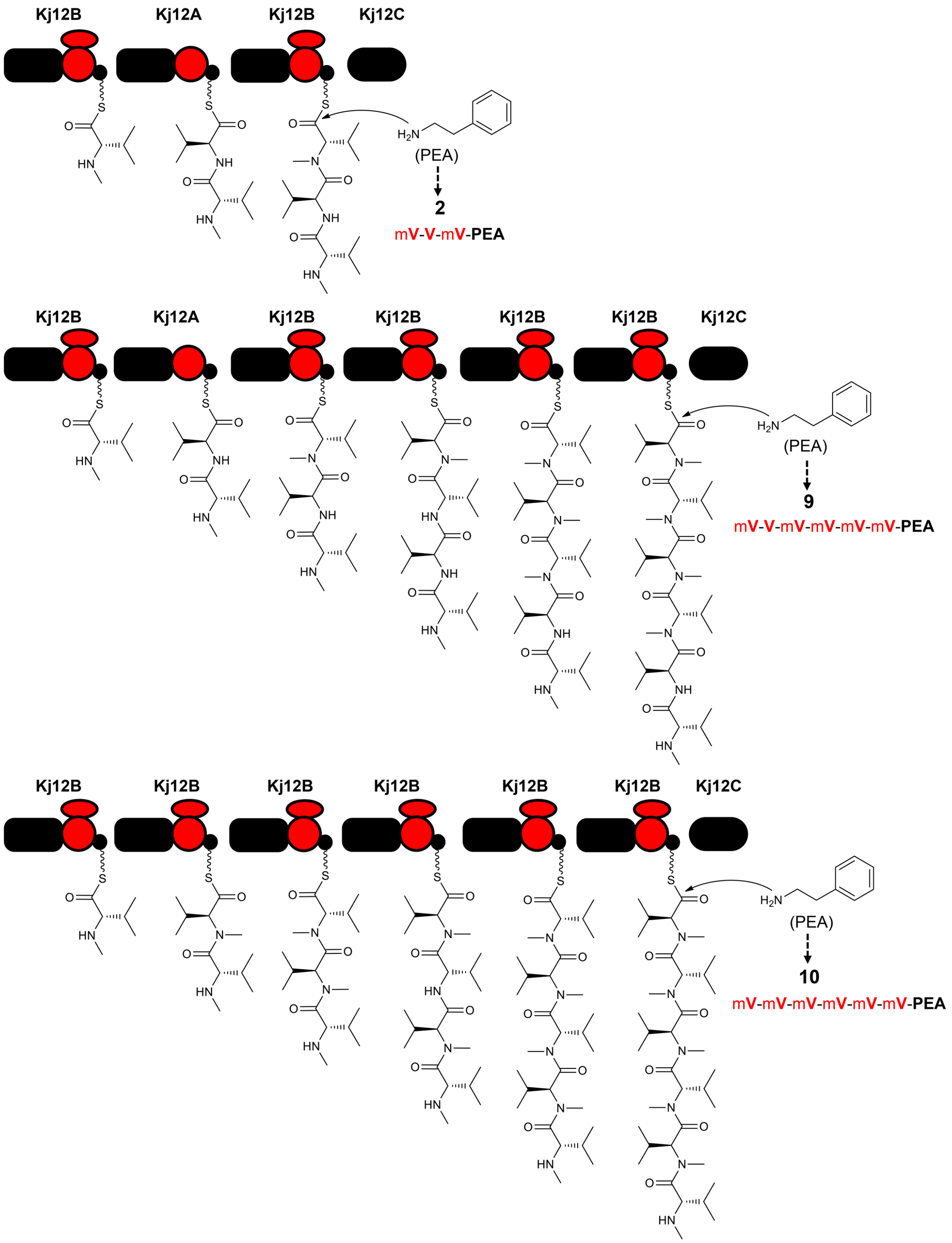

Supplementary Figure 1. Selected examples for proposed biosynthetic pathways of selected RXPs produced by Kj12B and Kj12C from *Xenorhabdus stockiae* KJ12.1.

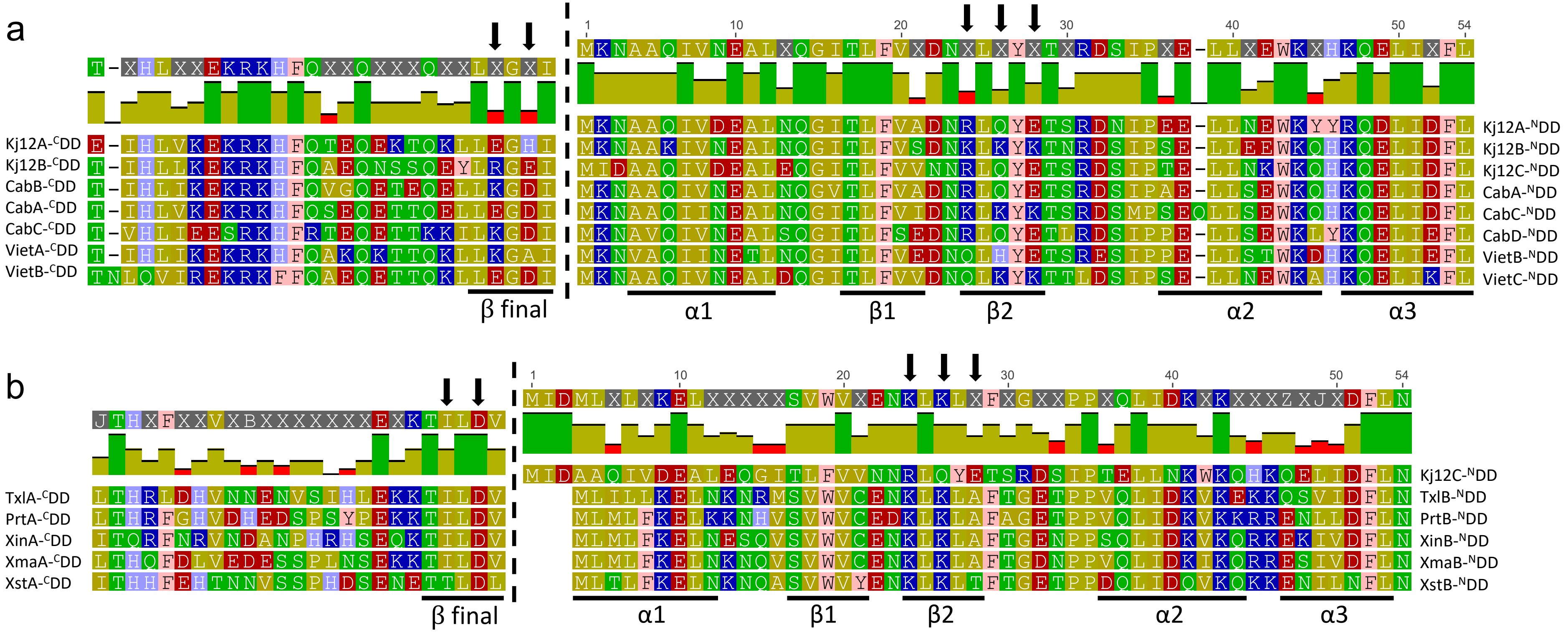

Supplementary Figure 2. Alignment of selected NRPS-^{C}DD and -^{N}DDs identified by BLASTP search using Kj12C-^{N}DD as query sequence. Secondary structural elements are indicated below. Arrows indicate key residue positions in charge of mediating ^{C}DD-to- ^{N}DD specific interaction as part of an anti-parallel β-sheet secondary structure arrangement. **a**, Alignment of Xenorhabdus RXP NRPS-DDs (CabCD, *X. cabanillasii*; VietABC, *X. vietnamensis*)[1] **b**, Alignment of closely related but non-RXP NRPS-^{C}DDs and -^{N}DDs from *Xenorhabdus* strains showing similar structures as Kj12C-^{N}DD connecting NRPS subunits carrying C-terminal epimerization (E) to N-terminal condensation (C) domains. PrtAB from *X. doucetiae*; Xma, *X. mauleonii*; Xin, *X. innexii*; and Xst, *X. stockiae*[2], TxlAB (TaxlIIaid)[3]. The consensus alignment is depicted above the alignment sequences with a ⩾75% threshold implemented. Alignments were performed using the multiple alignment program MUSCLE (default parameters)[4,5].

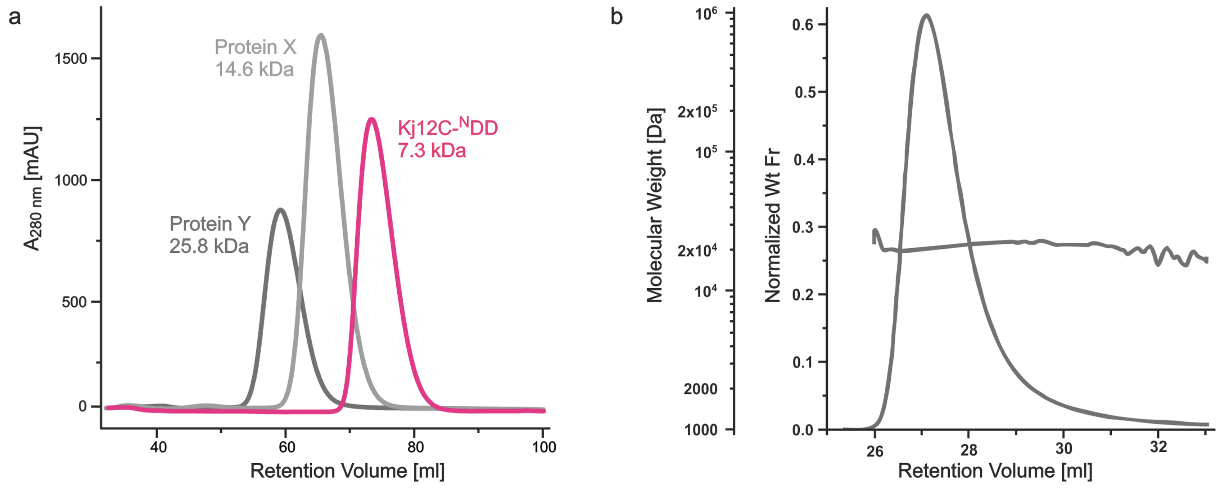

Supplementary Figure 3. Kj12C-^{N}DD is a monomeric protein. a, the different size exclusion chromatography runs on a HiPrep 16/60 Sephacryl S-100 column (GE Healthcare) in 50 mM NaPP pH6.5 with 100 mM NaCl. For comparison with the 7.3 kDa Kj12C-^{N}DD (magenta) two larger monomeric proteins were used (NisI$_{2\text{-}226}$ 25.8 kDa in dark gray and NisI$_{97\text{-}226}$ 14.6 kDa in light gray.[6] **b**, size exclusion chromatography with multi angle light scattering of Kj12C-^{N}DD in the same buffer as in (a) on a Superdex 75 10/300 GL column (GE Healthcare) with a calculated MW of 7.189 kDa.

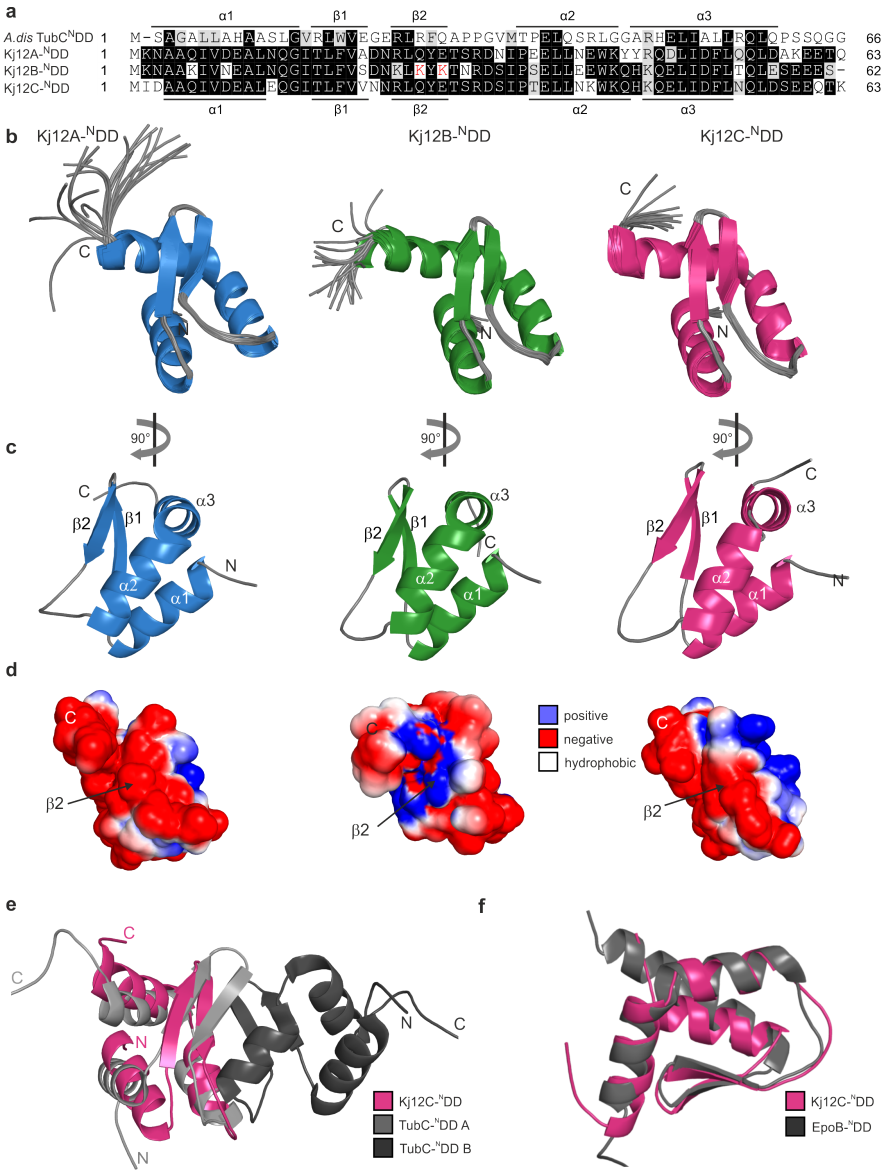

Supplementary Figure 4. N-terminal docking domains have the same three-dimensional structure. a, Structure based sequence alignment of the TubC-^{N}DD and the N-terminal docking domains of Kj12A-C. Identical residues are highlighted with *dark grey boxes* and residues with similar chemical properties are shown in *light grey boxes*. The secondary structure based on structural information for TubC-^{N}DD and Kj12C-^{N}DD are indicated *above* and *below* the sequence. **b,** Solution structure bundle of the 19 lowest energy conformers and the regularized mean structure for Kj12A-^{N}DD (blue), Kj12B-^{N}DD (green) and Kj12C-^{N}DD (magenta). **c,** Cartoon representations of the energy minimized mean structures of Kj12A-C ^{N}DDs with the same color-coding as in b rotated by 90°. **d,** Electrostatic surface potentials of each ^{N}DD mapped on the solvent accessible surfaces in the same orientation as in b with negatively charged surface areas coloured in *red*, positively charged areas coloured in *blue* and *white* areas corresponding to hydrophobic surfaces. **e,** Overlay of the energy minimized mean structure of Kj12-^{N}DD from this work (magenta) with one monomer of the TubC-^{N}DD structure (PDB code 2jug).[7] **f,** Overlay of the energy minimized mean structure of Kj12C-^{N}DD from this work (magenta) with the crystal structure of the monomeric EpoBcy with the N-terminal docking domain in light grey (PDB code 5t81 chain A).[8]

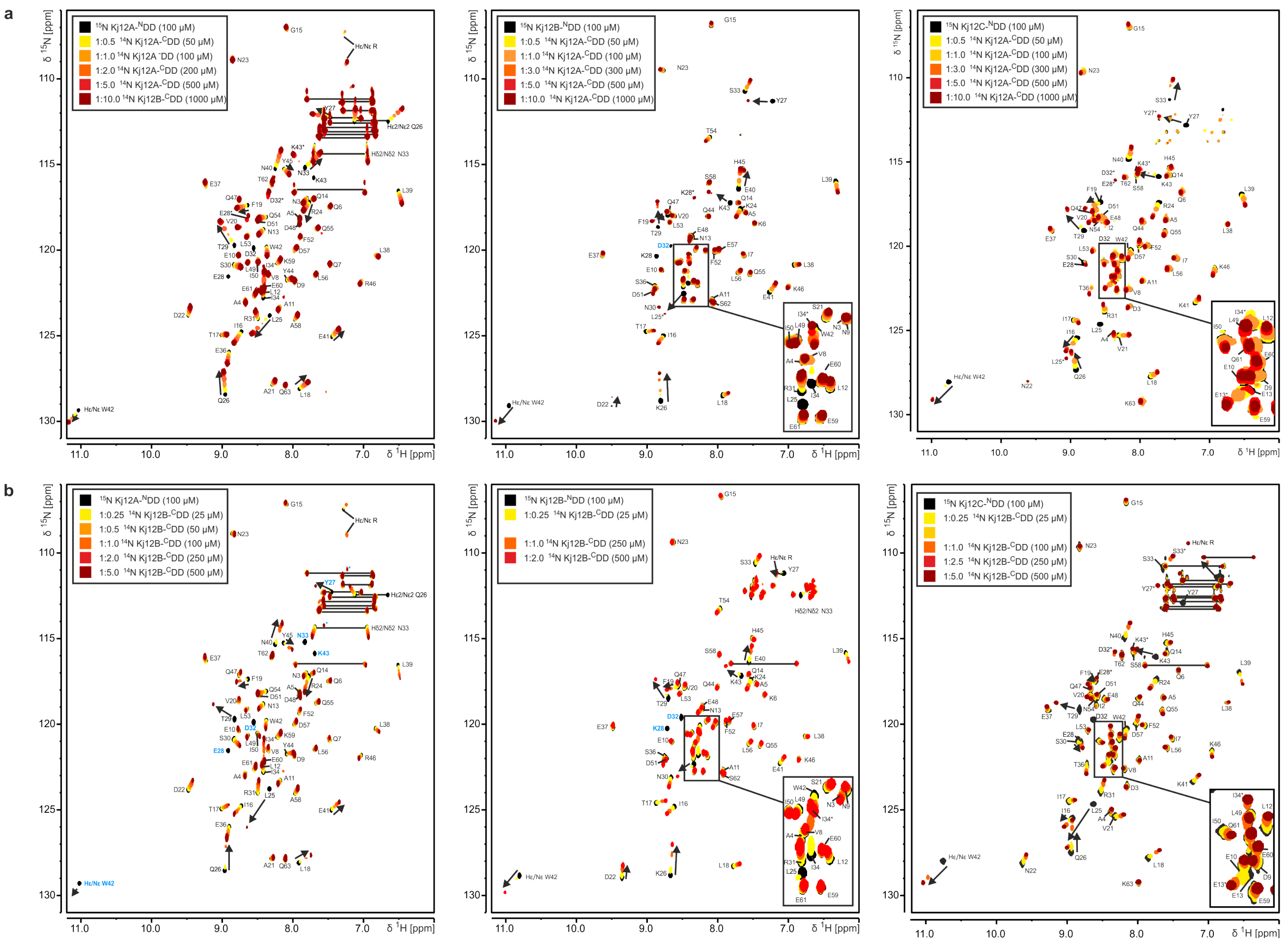

Supplementary Figure 5. ^{1}H, ^{15}N-HSQC spectra of docking domain titration. a, 100 µM ^{15}N labeled Kj12A-^{N}DD (left), Kj12B-^{N}DD (middle) and Kj12C-^{N}DD (right) with increasing amounts of ^{14}N Kj12A-^{C}DD (50 µM yellow, 100 µM light orange, 300 µM dark orange, 500 µM red, and 1000 µM dark red, not every step was recorded for all ^{N}DDs. Direction of peak shifting is indicated with arrows for some peaks. Possible bound state peaks are indicated with asterisk. **b,** 100 µM ^{15}N labeled Kj12A-^{N}DD (left), Kj12B-^{N}DD (middle) and Kj12C-^{N}DD (right) with increasing amounts of ^{14}N Kj12B-^{C}DD (25 µM yellow, 50 µM light orange, 100 µM dark orange, 250 µM red, and 500 µM dark red, not every step was recorded for all ^{N}DDs. The directions of peak shifts are indicated with arrows for some peaks. For Kj12A-^{N}DD and Kj12B-^{N}DD peaks in intermediate exchange are indicated with blue residue numbers for those peaks where the bound state could not be assigned unambigiously. Putative bound state peaks are indicated with asterisks. For Kj12C-^{N}DD all bound state peaks could be assigned in comparison with the spectrum of the two covalently linked domains (Kj12C-^{N}DD-12xGS-Kj12B-^{C}DD).

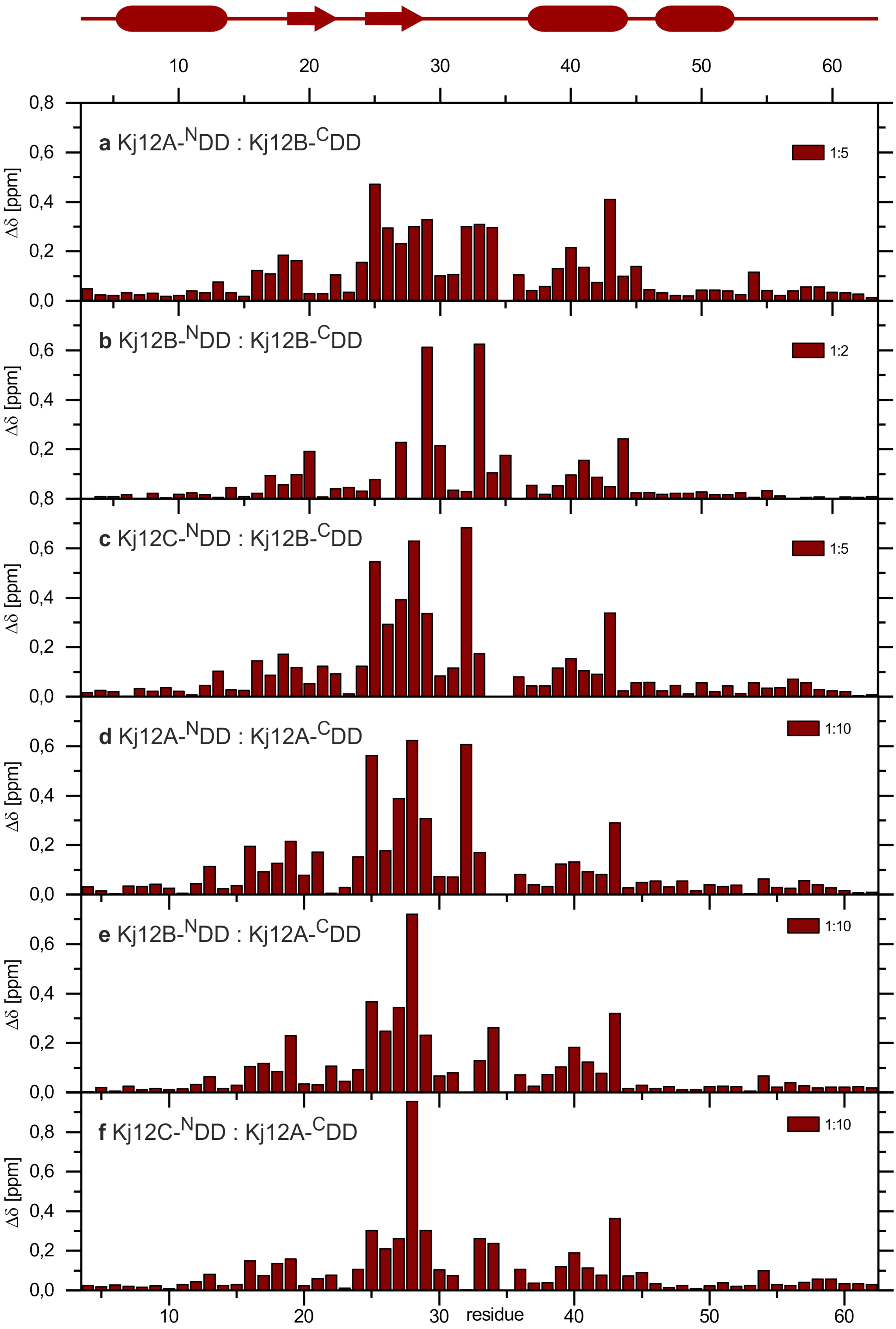

Supplementary Figure 6. Histogram of the chemical shift differences of the N-terminal docking domains of Kj12A, Kj12B and Kj12C upon addition of 5 equivalents of Kj12B-^{C}DD (**a-c**) and Kj12A-^{C}DD (**d-f**).

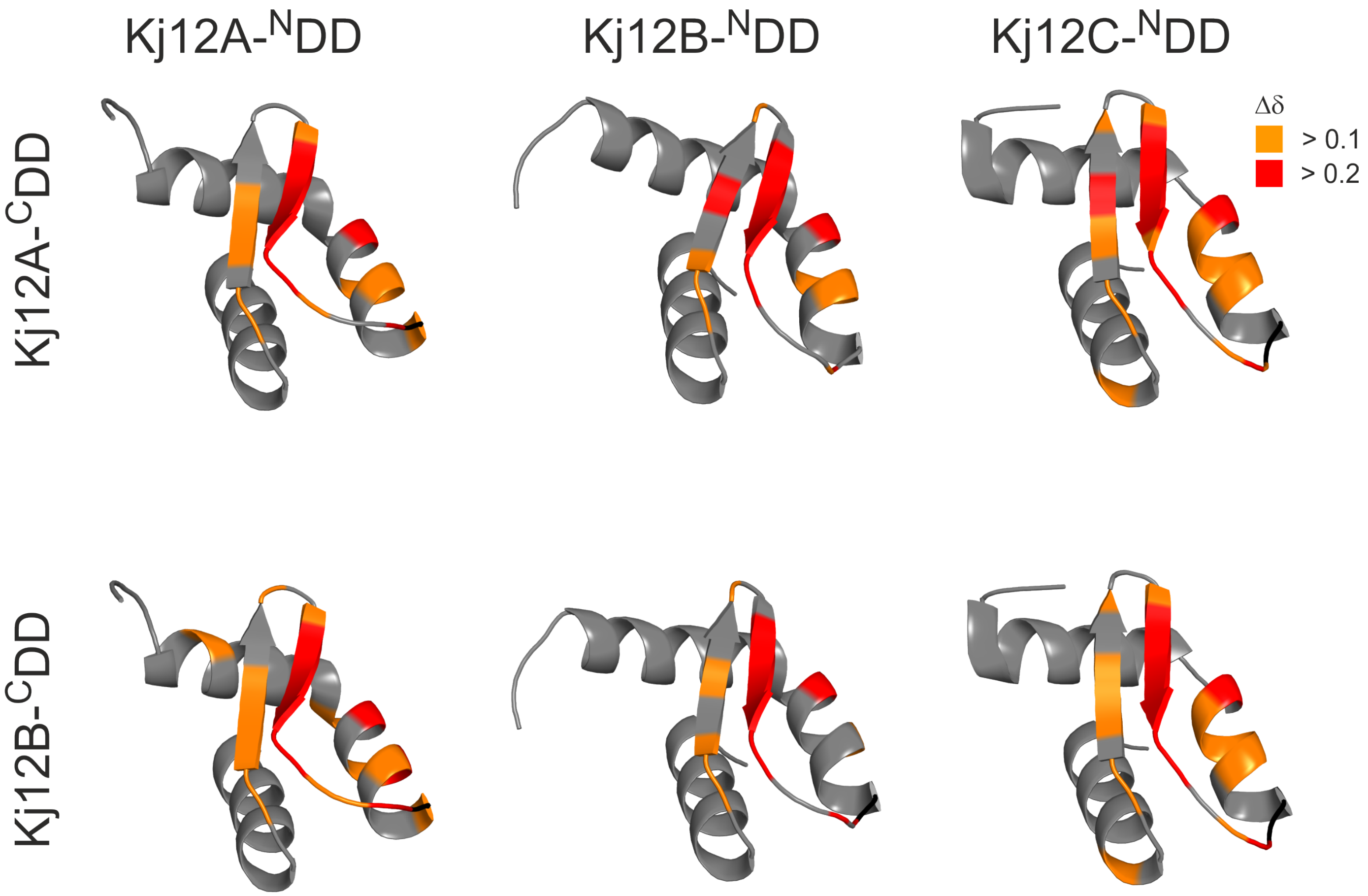

Supplementary Figure 7. Large chemical shift changes of docking domain titrations mapped on ^{N}DD structures. Large chemical shift changes upon addition of unlabeled Kj12A-^{C}DD in 10-fold excess mapped onto the structures of ^{N}DDs (upper panel) and of Kj12B-^{C}DD in 5-fold excess mapped onto the structures of ^{N}DDs (lower panel), unassigned residues are indicated in black.

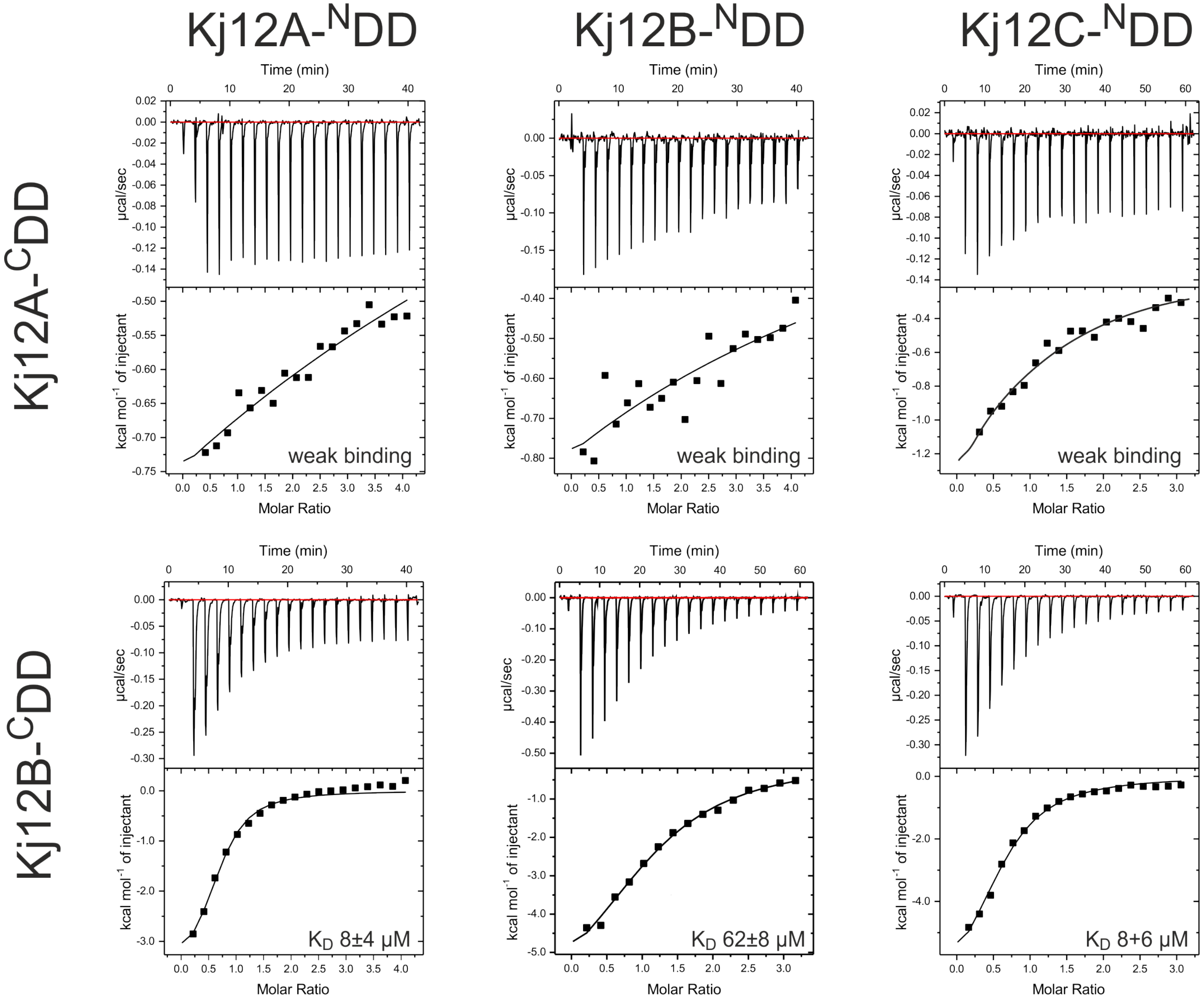

Supplementary Figure 8. Docking domain titrations with ITC. ITC thermograms and the derived binding curves for titrations of 50 □M ^{N}DDs in 50 mM $NaPP_i$ pH 6.5, 100 mM NaCl with ^{C}DDs. The K_ds for Kj12A-^{C}DD could not be measured reliably with ITC due to the low binding affinities (upper panel). The K_ds for Kj12B-^{C}DD were measured in triplicates (lower panel).

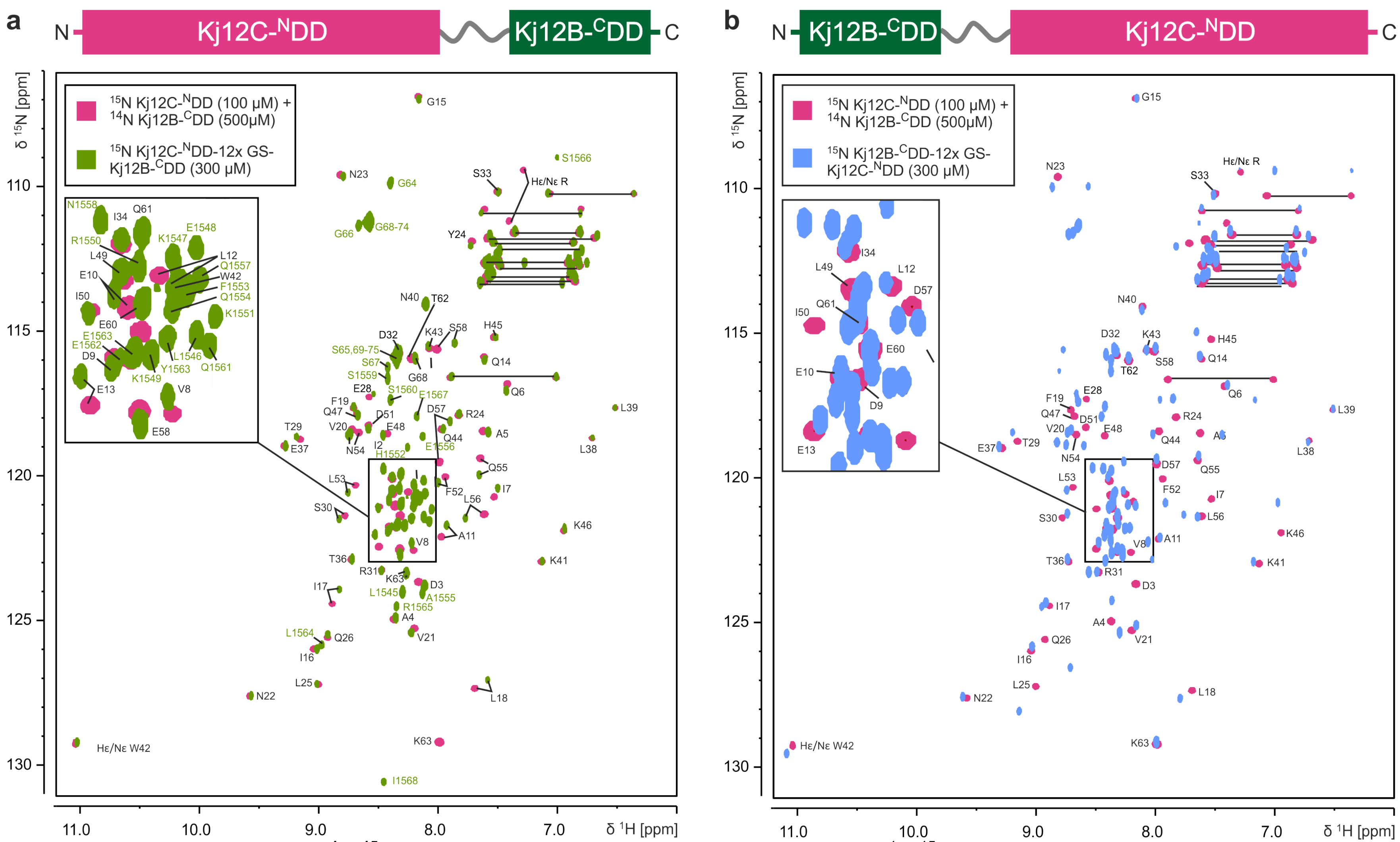

Supplementary Figure 9. Comparison of ^{1}H,^{15}N-HSQC spectra of different linker constructs. ^{1}H,^{15}N-HSQC spectra of titration endpoint (ratio 1:5) of Kj12C-^{N}DD with Kj12B-^{C}DD (red) overlaid with Kj12C-^{N}DD-12xGS-Kj12B-^{C}DD (green) in (**a**) and Kj12B-^{C}DD-12xGS-Kj12C-^{N}DD (blue) in (**b**). Schematic representations of linker constructs are indicated above.

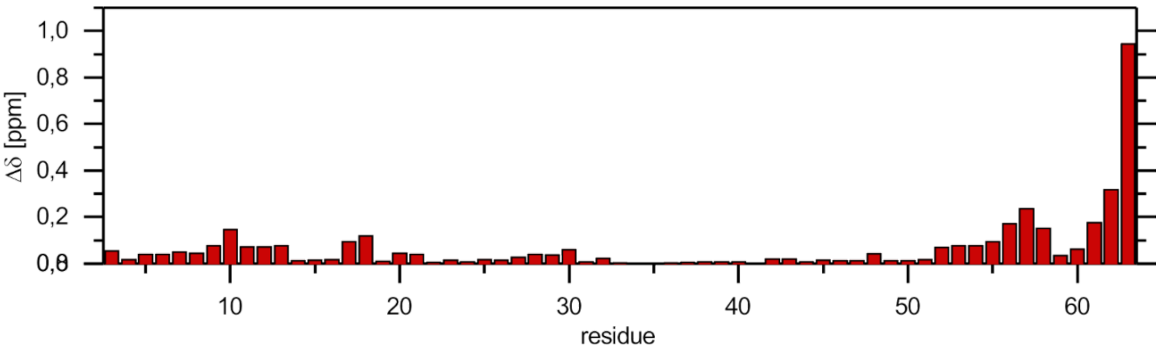

Supplementary Figure 10. Histogram of the chemical shift differences. Comparison of the $^{1}H,^{15}N$ HSQC spectra of the N-terminal docking domain of Kj12C bound to Kj12B-^{C}DD in trans (1:5 ratio, titration endpoint) and the covalently linked domains (Kj12C-^{N}DD-12xGS-Kj12B-^{C}DD).

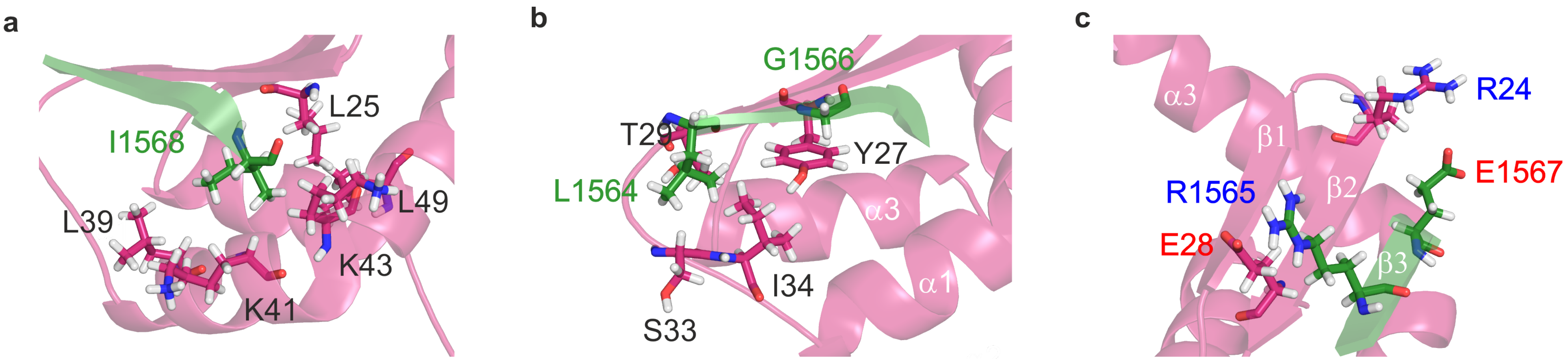

Supplementary Figure 11. Key residues of the ^{N}DD-^{C}DD complex. Complex structure of Kj12C ^{N}DD (magenta) and Kj12B ^{C}DD (green) with structural important residues are shown in stick representation. **a**, I1568 of Kj12B-^{C}DD is buried in a hydrophibic pocket formed by residues of helix α2 and α3. **b**, L1564 of Kj12B-^{C}DD is buried in a hydrophibic pocket formed by residues of loop 2 and Y27 stacks under G1566. **c**, Two salt bridges are formed between R24 and E1567 and between E28 and R1565.

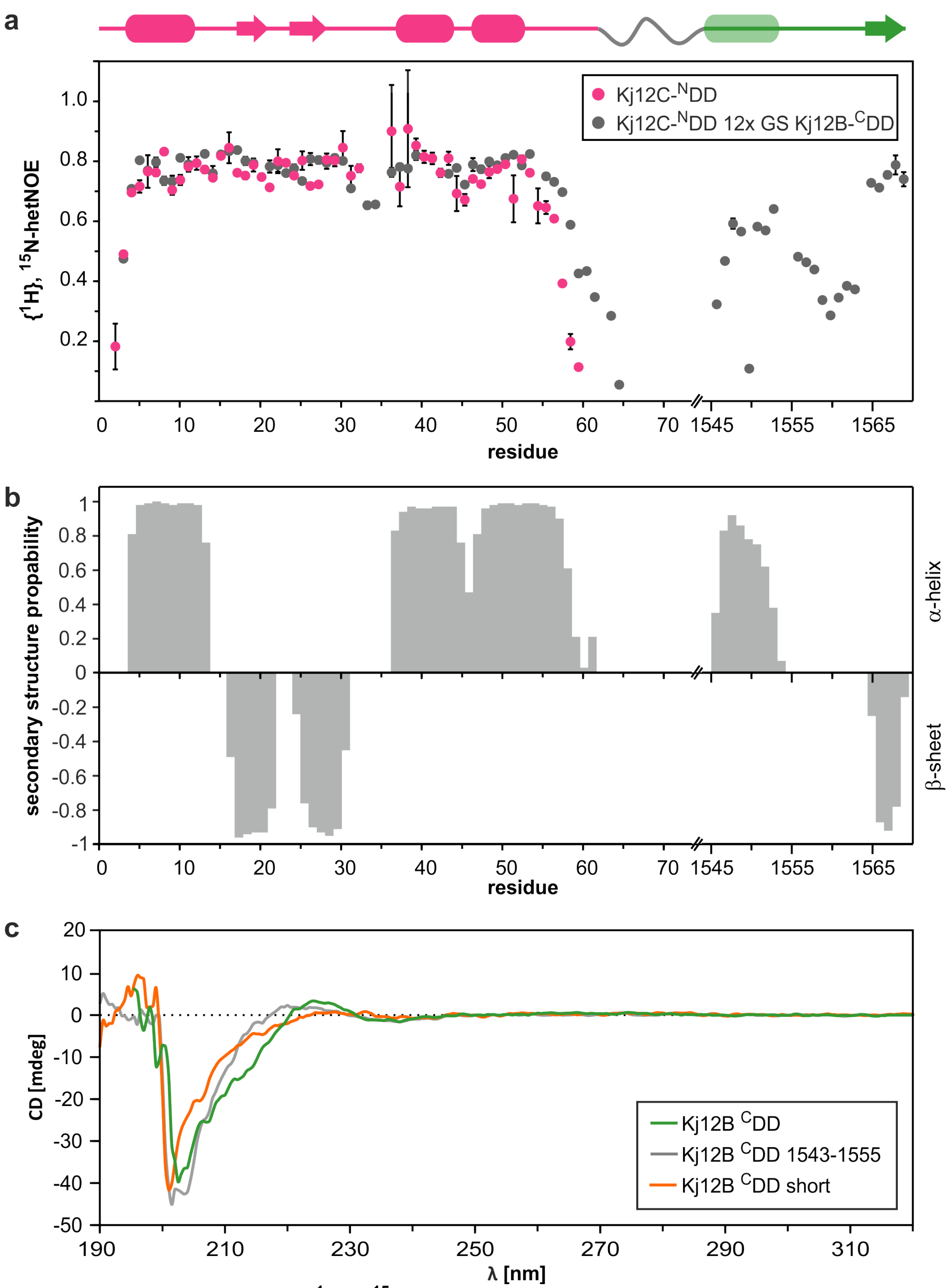

Supplementary Figure 12. ^{1}H, ^{15}N-hetNOE measurements, secondary structure probability and CD measurements. a, Values of hetNOE measurements of Kj12C-^{N}DD 12xGS Kj12B-^{C}DD complex (grey) overlayed with free Kj12C-^{N}DD (magenta) plotted onto the sequence. Secondary structure elements derived from structure calculation are indicated ontop. **b**, Secondary structure propability of the Kj12C-^{N}DD 12xGS Kj12B-^{C}DD complex as derived from backbone chemical shifts using the program TalosN. **c**, CD spectroscopic analysis of Kj12B-^{C}DD, Kj12B-^{C}DD 1543-1555 – a peptide corresponding to the transient α-helix in the complex structure - and Kj12B-^{C}DD$_{short}$. All three peptides show characteristics for a random coil or unstructrued conformation in the CD spectrum with a strog negative peak at 200 nm.[9]

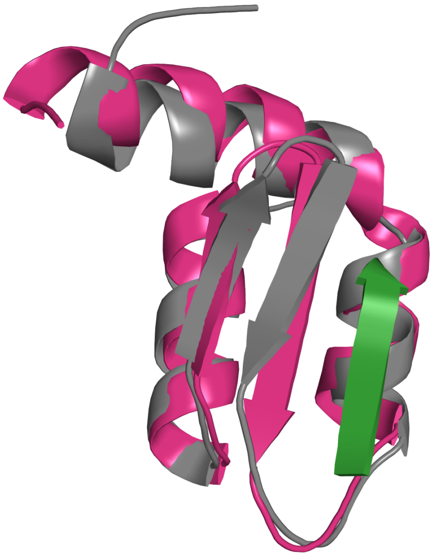

Supplementary Figure 13. Structure overlay of Kj12C-^{N}DD free and bound. Overlay of the mean structures of Kj12C ^{N}DD alone in gray with the complex structure of Kj12C-^{N}DD-12xGS-Kj12B-^{C}DD (residues 1-63 in magenta and residues 1564-1568 in green, flexible residues are not shown). Residues 1-63 align with a Cα-RMSD of 1.18 Å.

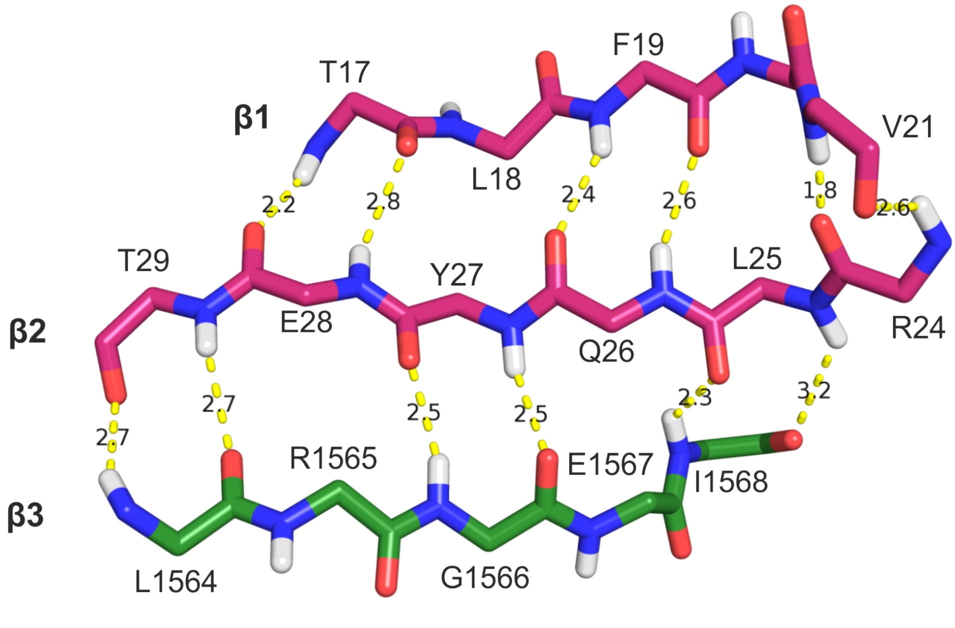

Supplementary Figure 14. Hydrogens bonds in the ^{N}DD-^{C}DD complex across the intermolecular β-sheet. Detailed view of the ^{N}DD-^{C}DD interaction of β-sheet 2 of Kj12C-^{N}DD (magenta) with the last 5 amino acids of Kj12B-^{C}DD (green), hydrogen bonds are shown in stick representation with the N-C distances given in Ångström.

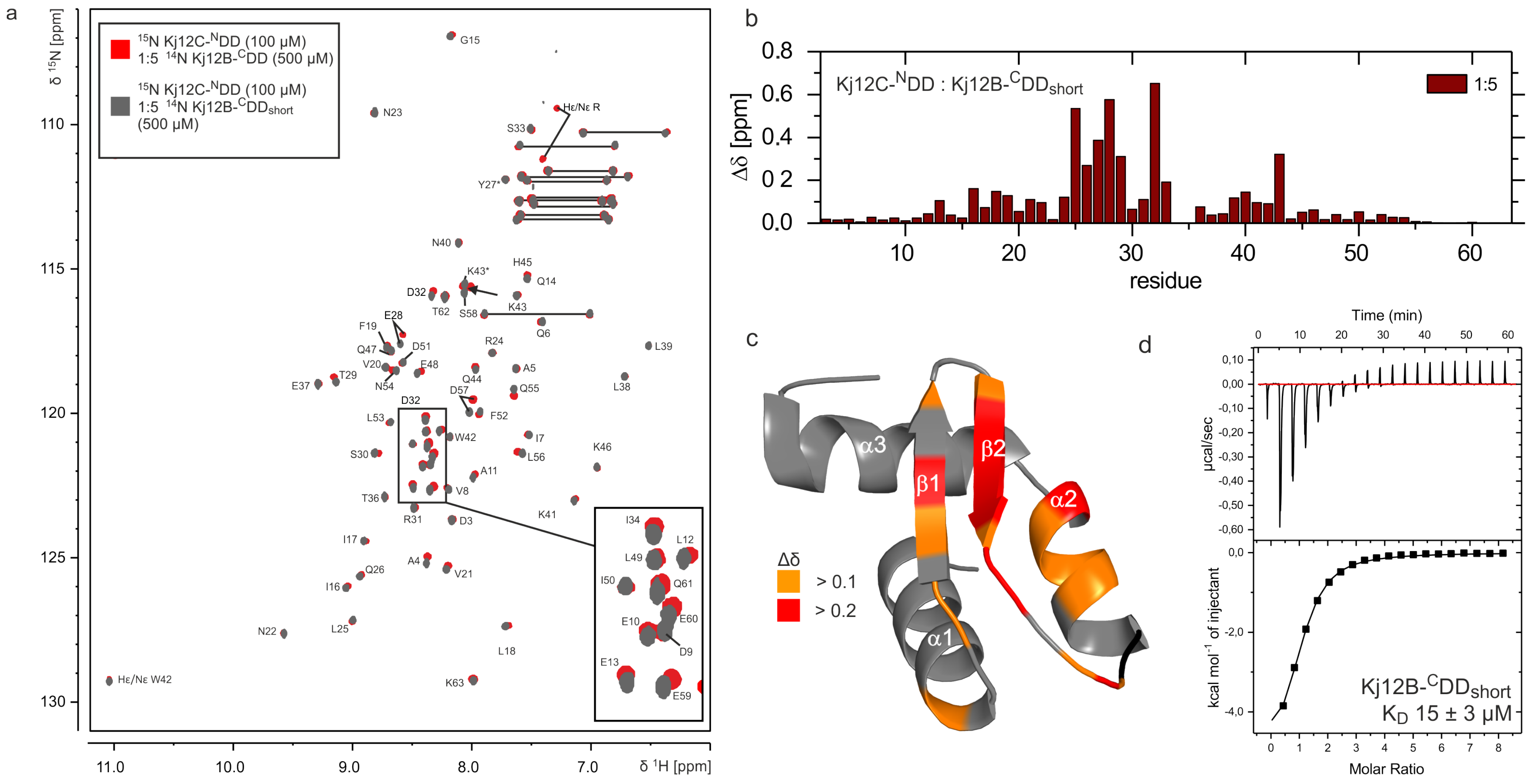

Supplementary Figure 15. Titration with Kj12B-^{C}DD$_{short}$. a, ^{1}H, ^{15}N-HSQC spectra for titrations of 100 µM ^{15}N labeled Kj12C-^{N}DD with a 5-fold excess of unlabeled Kj12B-^{C}DD (red) and 100 µM ^{15}N labeled Kj12C-^{N}DD with a 5-fold excess of unlabeled Kj12B-^{C}DD$_{short}$ (gray). **b**, Histogram of the chemical shift differences of the ^{1}H,^{15}N HSQC spectra of the free N-terminal docking domain of Kj12C and bound to Kj12B-^{C}DD$_{short}$ (1:5 ratio, titration endpoint). **c**, Chemical shift changes upon addition of unlabeled Kj12B-^{C}DD$_{short}$ in 5-fold excess mapped onto the structure of the ^{N}DD. **d**, ITC thermograms and the derived binding curves for titrations of Kj12C-^{N}DD with Kj12B-^{C}DD$_{short}$.

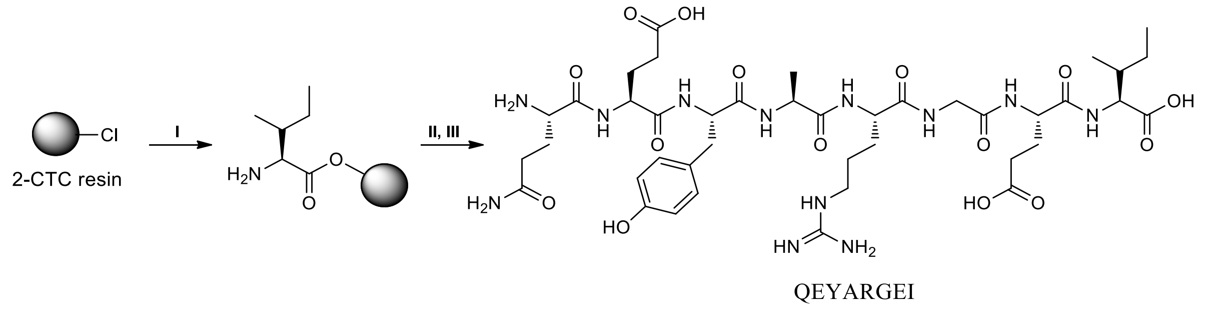

Supplementary Figure 16. Representative solid phase synthesis of $^{C}DD_{short}$ peptide derivatives exemplarily shown for QEYARGEI. I, Fmoc-Ile-OH, DIPEA, DCM, overnight, DCM/CH_3OH/DIPEA (80:15:5), then piperidine/NMP. **II**, Fmoc-AA-OH, HCTU, DMF, DIPEA, NMP, 50 min, then piperidine/NMP. **III**, TFA/TIS/water (95:2.5:2.5), 3 h.

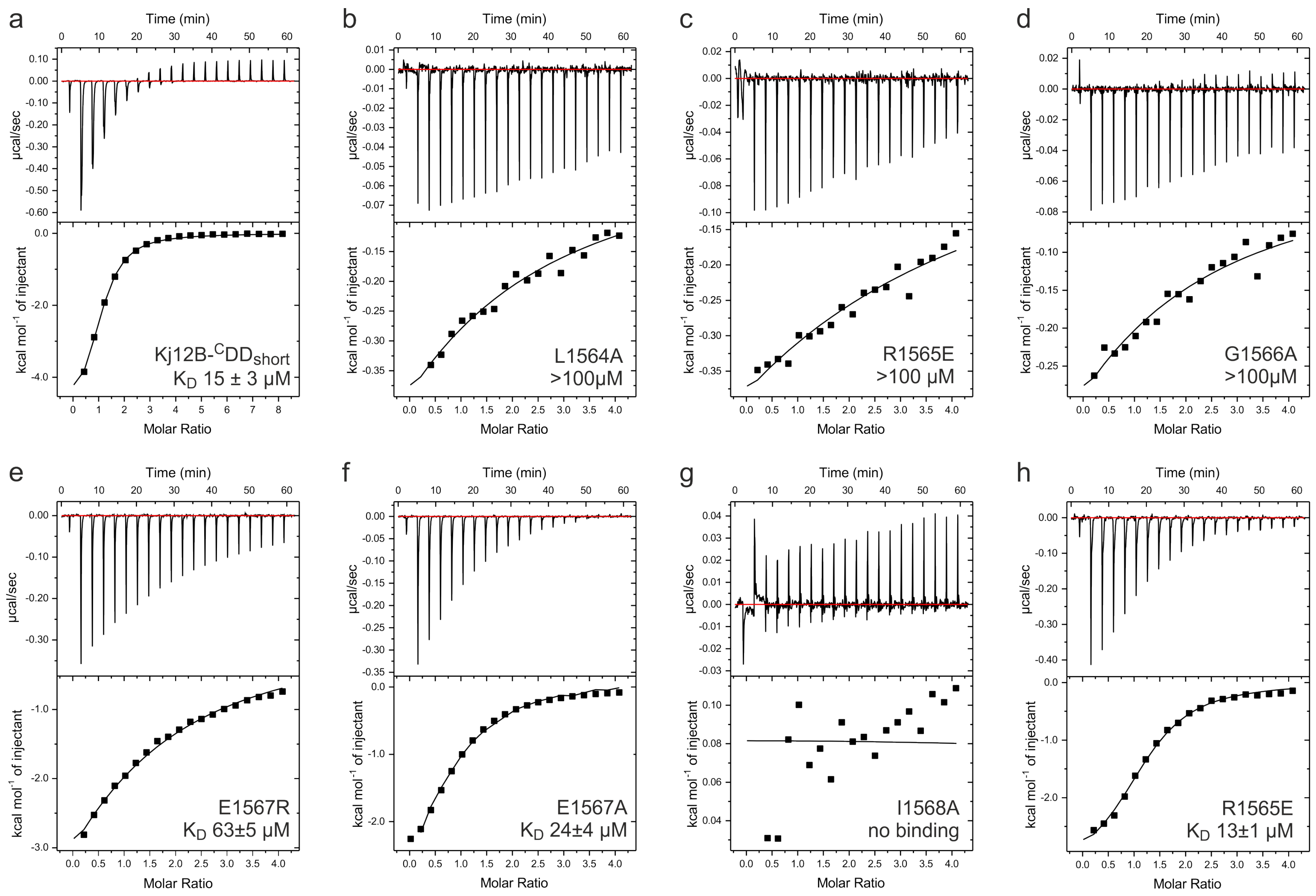

Supplementary Figure 17. ITC titration with variants of Kj12B-^{C}DD$_{short}$. a, Titration of Kj12C-^{N}DD with the native Kj12B-^{C}DD$_{short}$ peptide **b-g**, Titration of Kj12C-^{N}DD with variants of the Kj12B-^{C}DD$_{short}$ peptide (L1564A, R1465E, G1566A, E1567R, E1567A and I1568A). **h**, Titration of Kj12B-^{N}DD with Kj12B-^{C}DD$_{short}$ R1565E peptide.

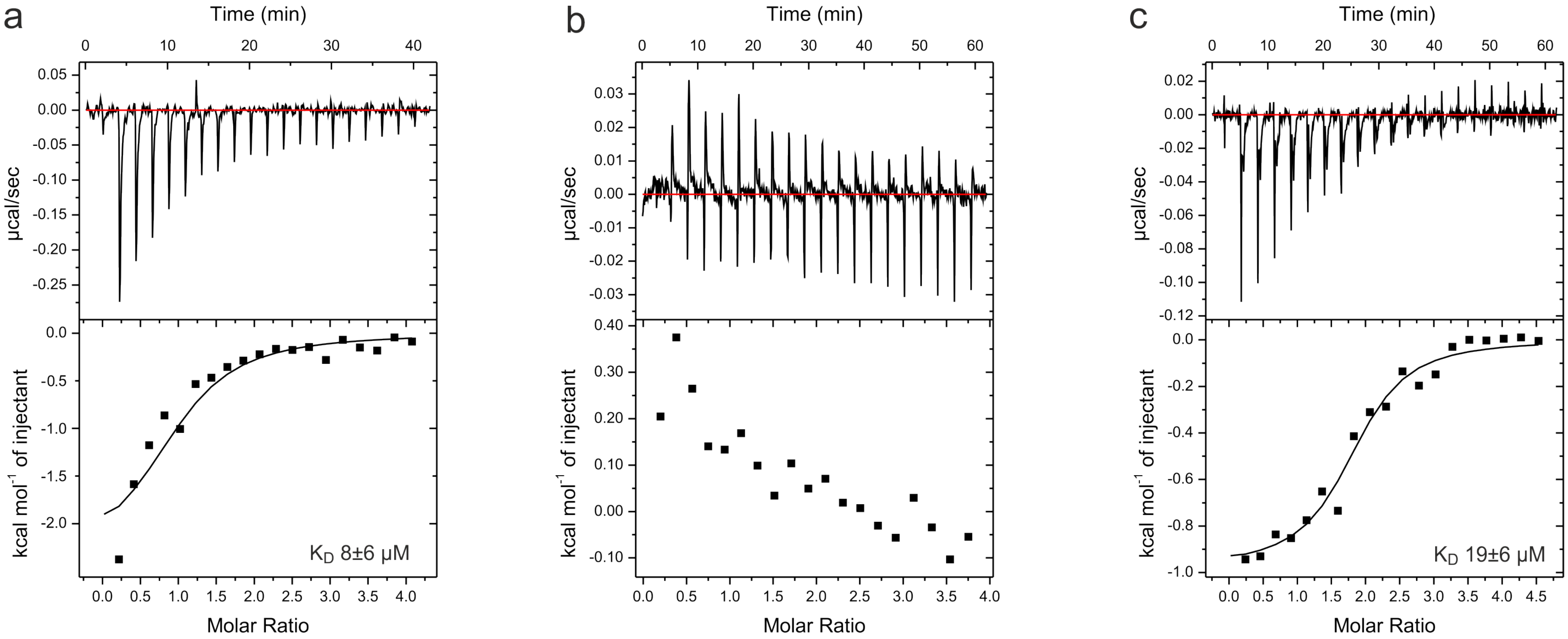

Supplementary Figure 18. ITC titration of docking domains with adjacent domains Titration of **a** Kj12C-^{N}DD-Cterm and **b** Kj12C-Cterm without the N-terminal docking domain with the Kj12B-$^{C}DD_{short}$ peptide. **c**, Titration of Kj12B-Tdom-^{C}DD with the Kj12C-^{N}DD.

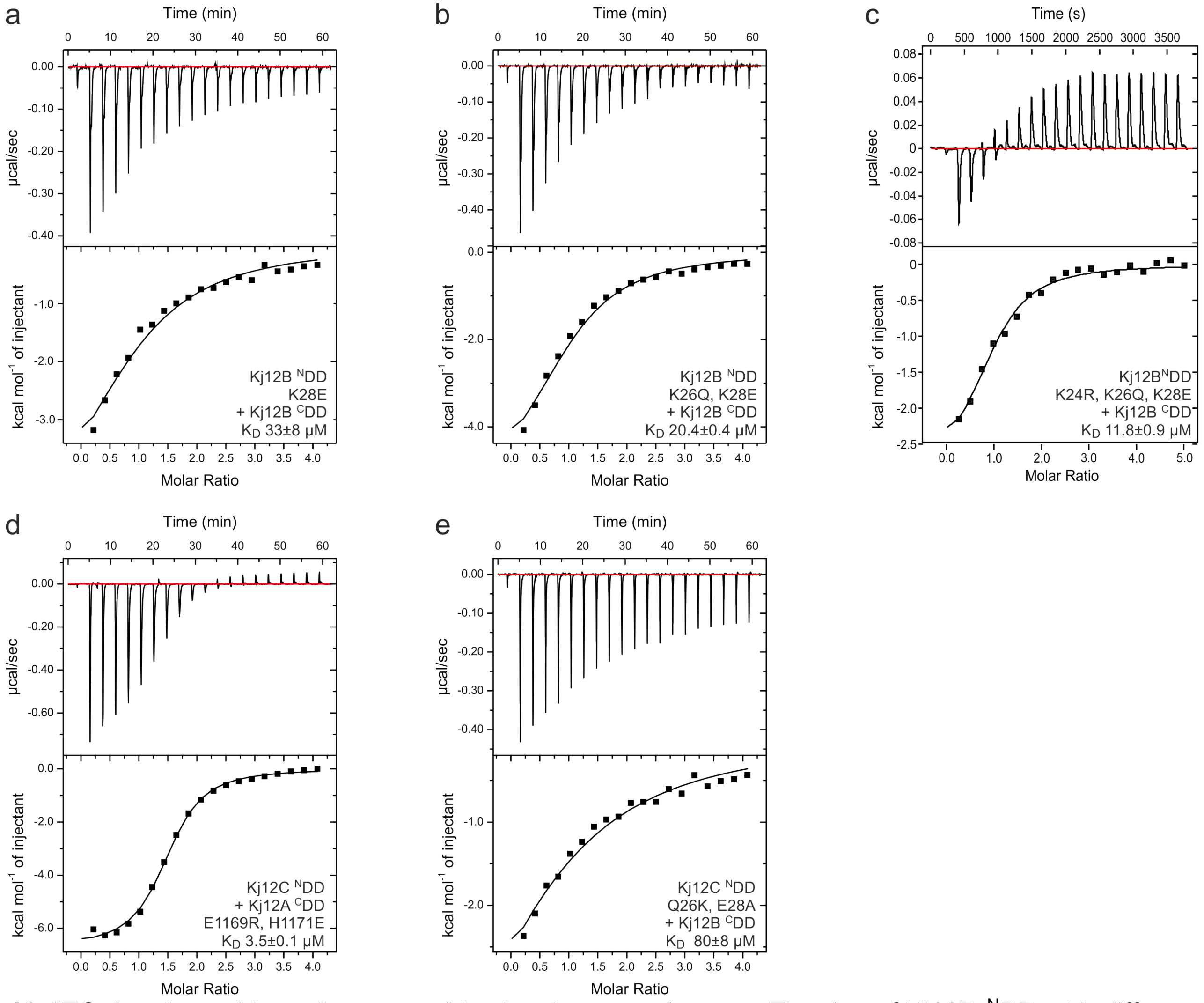

Supplementary Figure 19. ITC titration with variants used by *in vivo* experiments. Titration of Kj12B-^{N}DD with different point mutations with the Kj12B-^{C}DD$_{short}$ peptide. The single mutant of Kj12B ^{N}DD (K28E) is shown in **a**, the double mutant (K26Q, K28E) in **b** and the triple mutant (K24R, K26Q, K28E) in **c**; **d** Kj12C ^{N}DD titrated with optimized Kj12A ^{C}DD$_{short}$ E1169R, H1171E; **e** Kj12C ^{N}DD Q26E, E28A titrated with Kj12B ^{C}DD$_{short}$.

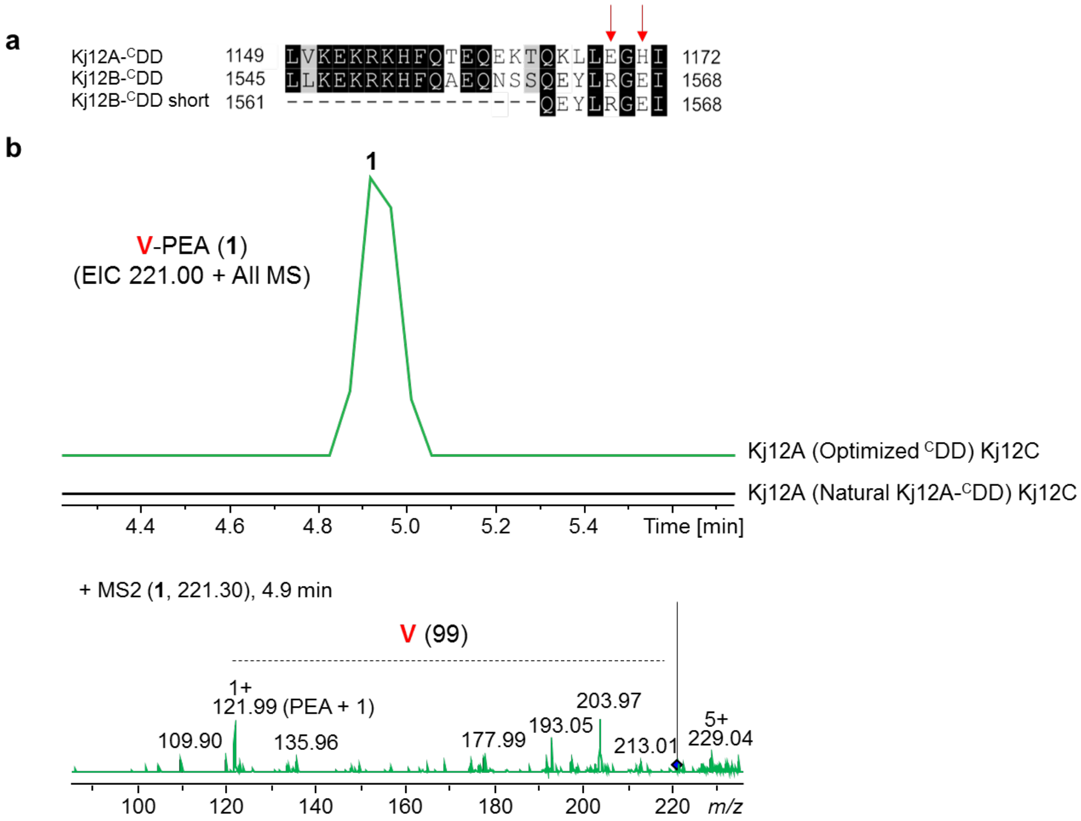

Supplementary Figure 20. Optimization of Kj12A-^{C}DD facilitated the interaction between Kj12A and Kj12C, leading to the production of V-PEA (1). a, Sequence alignment of ^{C}DDs of Kj12A and Kj12B. The essential interactive motif of Kj12B-^{C}DD was shown as Kj12B-$^{C}DD_{short}$. The key residues on the motif were indicated with red arrows. **b**, EIC of **1** produced in the coexpression system of modified Kj12A and natural Kj12C, relative to that in the natural Kj12A and Kj12C. MS/MS fragmentation of **1** is also shown.

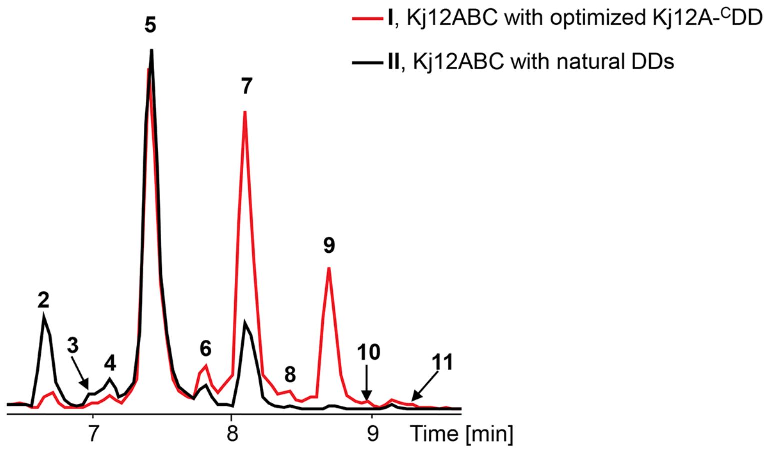

Compound	Rt/min	*m/z*	Structure	Relative RXP amount	
				I	II
**2**	6.7	447.3	mV-V-mV-PEA	5.6 ± 0.3	20.7 ± 8.6
**3**	6.9	532.3	V-V-V-mV-PEA	0.9 ± 0.5	0.5 ± 0.9
**4**	7.3	546.4	mV-V-mV-V-PEA	8.4 ± 2.6	15.8 ± 15.8
**5**	7.4	560.3	mV-V-mV-mV-PEA	100.0 ± 10.0	100.0 ± 10.0
**6**	7.8	574.4	mV-mV-mV-mV-PEA	5.5 ± 0.7	5.1 ± 10.3
**7**	8.1	673.3	mV-V-mV-mV-mV-PEA	93.3 ± 8.4	28.2 ± 16.1
**8**	8.4	687.3	mV-mV-mV-mV-mV-PEA	7.2 ± 3.0	2.6 ± 2.8
**9**	8.7	786.3	mV-V-mV-mV-mV-mV-PEA	44.1 ± 4.5	3.2 ± 4.3
**10**	9	800.4	mV-mV-mV-mV-mV-mV-PEA	3.5 ± 0.6	0.5 ± 0.2
**11**	9.3	899.5	mV-V-mV-mV-mV-mV-mV-PEA	2.5 ± 0.8	–

Supplementary Figure 21. Comparison of RXPs produced in natural and optimized Kj12ABC systems via LC/MS analysis. Base peak chromatograms (BPCs) from both systems were shown. Optimized Kj12ABC (I), red line; natural Kj12ABC (II), black line. The table shows the retention time (Rt), detected masses (*m/z*) in [M+H]+, structures (simplified codes) and relative production of all RXPs produced in both systems as determined from triplicate experiments.

a

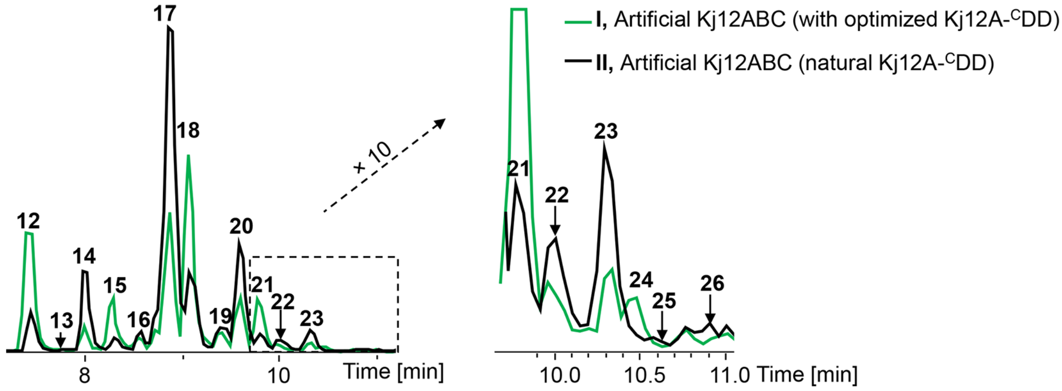

Compound	Rt/min	*m/z*	Structure	Relative RXP amount	
				I	II
**12**	7.5	475.5	V-mL-mL-PEA	72.3 ± 9.1	10.0 ± 1.3
**13**	7.9	489.4	mL-L-mL-PEA	1.31 ± 0.5	1.6 ± 2.7
**14**	8.0	503.4	mL-mL-mL-PEA	10.8 ± 4.2	20.3 ± 2.4
**15**	8.3	602.4	V-mL-mL-mL-PEA	29.4 ± 4.5	4.6 ± 1.3
**16**	8.7	616.5	mL-L-mL-mL-PEA	10.1 ± 2.2	12.9 ± 11.6
**17**	8.8	630.1	mL-mL-mL-mL-PEA	62.7 ± 15.2	100.0 ± 10.0
**18**	9.1	729.1	V-mL-mL-mL-mL-PEA	100.0 ± 10.0	25.6 ± 2.5
**19**	9.4	743.5	mL-L-mL-mL-mL-PEA	12.5 ± 2.2	11.3 ± 3.2
**20**	9.6	757.5	mL-mL-mL-mL-mL-PEA	21.2 ± 5.7	32.0 ± 3.8
**21**	9.8	856.6	V-mL-mL-mL-mL-mL-PEA	20.5 ± 6.5	6.0 ± 0.6
**22**	10.2	870.4	mL-L-mL-mL-mL-mL-PEA	2.6 ± 0.8	1.9 ± 0.7
**23**	10.3	884.7	mL-mL-mL-mL-mL-mL-PEA	3.3 ± 1.4	6.6 ± 0.7
**24**	10.5	983.7	V-mL-mL-mL-mL-mL-mL-PEA	2.6 ± 0.9	0.9 ± 0.1
**25**	10.7	997.7	mL-L-mL-mL-mL-mL-mL-PEA	0.08 ± 0.02	0.1 ± 0.1
**26**	11.0	1011.6	mL-mL-mL-mL-mL-mL-mL-PEA	0.3 ± 0.08	0.5 ± 0.2

b

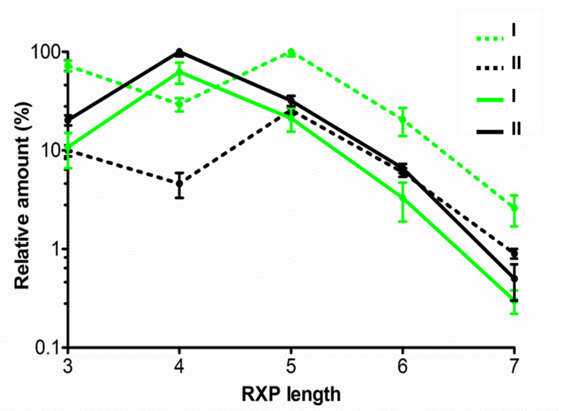

Supplementary Figure 22. Comparison of RXPs produced in artificial Kj12ABC. (Val specific A-MT from Kj12B was replaced against that from VietB for Leu specificity) with natural Kj12A-CDD (black, II) and optimized Kj12A-CDD (green, I) analyzed by HPLC/MS. **a**, BPCs from both systems were shown. The table shows the retention time (Rt), detected masses (*m/z*) in $[M+H]^+$, structures (simplified codes: Val, V; Leu, L; *N*-methylated Leu, mL) and relative production of all RXPs produced in both systems as determined from triplicate experiments. **b**, The diagram shows the relative amounts (%) and peptide length (in numbers of amino acids) of fully methylated RXPs (solid line) and RXPs containing one Val (dash line) produced in both systems.

a

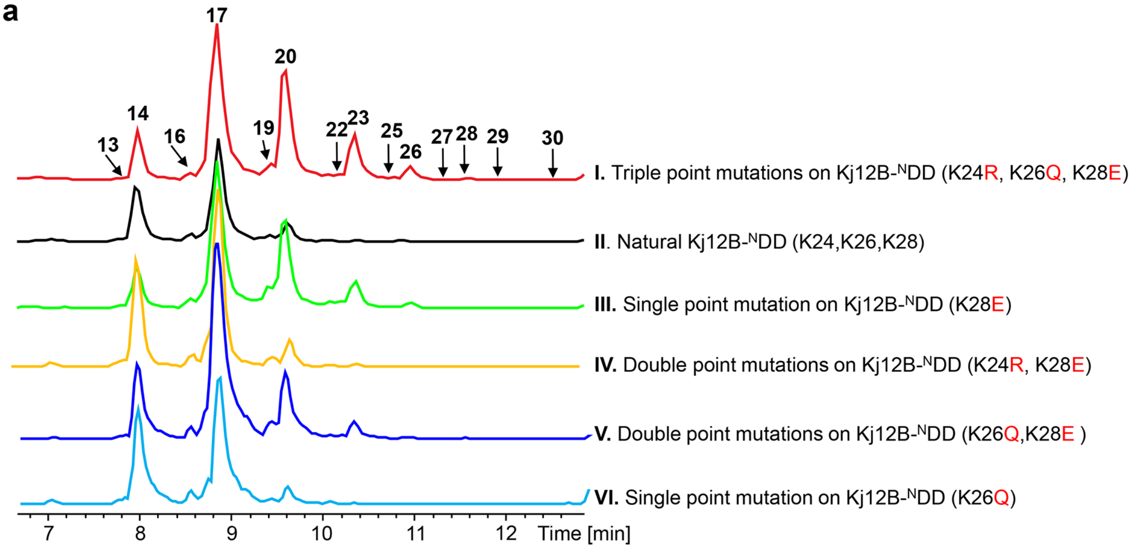

Compound	Rt/min	*m/z*	Structure	Relative RXP amount					
				I	II	III	IV	V	VI
**13**	7.9	489.4	mL-L-mL-PEA	1.8 ± 0.4	4.3 ± 1.3	2.8 ± 0.4	4.3 ± 0.7	2.6 ± 0.3	4.9 ± 5.5
**14**	8.0	503.4	mL-mL-mL-PEA	26.3 ± 1.3	53.6 ± 6.7	21.7 ± 1.3	49.5 ± 9.1	35.4 ± 0.04	69. ± 144.1
**16**	8.7	616.5	mL-L-mL-mL-PEA	74.8 ± 0.6	11.7 ± 4.0	15.6 ± 0.6	1.3 ± 1.5	12.1 ± 0.5	13.1 ± 46.4
**17**	8.8	630.1	mL-mL-mL-mL-PEA	100.0 ± 10.0	100.0 ± 10.0	100.0 ± 10.0	100.0 ± 10.0	100.0 ± 10.0	100.0 ± 10.0
**19**	9.4	743.5	mL-L-mL-mL-mL-PEA	13.0 ± 0.9	7.7 ± 2.8	17.0 ± 1.6	6.6 ± 1.5	9.1 ± 0.9	4.6 ± 9.4
**20**	9.6	757.5	mL-mL-mL-mL-mL-PEA	57.7 ± 5.5	18.6 ± 10.0	43.9 ± 11.2	14.0 ± 1.3	26.5 ± 5.8	10.8 ± 28.4
**22**	10.2	870.4	mL-L-mL-mL-mL-mL-PEA	3.0 ± 0.7	1.5 ± 1.5	4.0 ± 1.3	0.7 ± 0.1	1.5 ± 0.5	0.3 ± 1.3
**23**	10.3	884.7	mL-mL-mL-mL-mL-mL-PEA	21.5 ± 9.0	2.1 ± 1.6	14.4 ± 4.7	1.5 ± 0.2	5.3 ± 2.0	0.8 ± 3.7
**25**	10.7	997.7	mL-L-mL-mL-mL-mL-mL-PEA	0.5 ± 0.2	0.06 ± 0.1	0.4 ± 0.2	0.07 ± 0.02	0.4 ± 0.2	0.0 ± 0.2
**26**	11.0	1012.3	mL-mL-mL-mL-mL-mL-mL-PEA	0.4 ± 0.2	0.11 ± 0.1	0.1 ± 0.06	0.06 ± 0.03	–	–
**27**	11.3	1125.2	mL-L-mL-mL-mL-mL-mL-mL-PEA	0.05 ± 0.02	–	0.06 ± 0.02	–	–	–
**28**	11.5	1139.3	mL-mL-mL-mL-mL-mL-mL-mL-PEA	0.07 ± 0.02	–	0.03 ± 0.01	–	–	–
**29**	11.9	1266.1	mL-mL-mL-mL-mL-mL-mL-mL-mL-PEA	0.05 ± 0.08	–	–	–	–	–
**30**	12.3	1393.3	mL-mL-mL-mL-mL-mL-mL-mL-mL-mL-PEA	0.02 ± 0.004	–	–	–	–	–

b

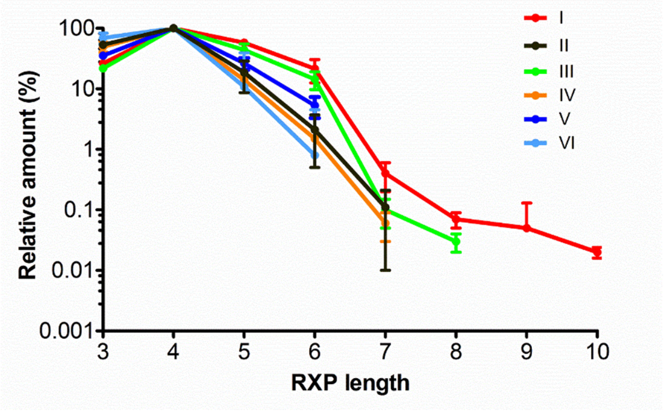

Supplementary Figure 23. Comparison of RXPs produced in artificial Kj12BC. (Val specific A-MT from Kj12B was replaced against that from VietB for Leu specificity) with natural Kj12B-^{N}DD (black, II) and optimized Kj12B-^{N}DDs: (K24R, K26Q, K28E) (red, I); (K28E) (green, III); (K24R, K28E) (yellow, IV); (K26R, K28E) (sapphire, V); and (K26Q) (light blue, VI) via LC/MS analysis. **a**, BPCs from all systems were shown. The table shows the retention time (Rt), detected masses (*m/z*) in $[M+H]^+$, structures (simplified codes, Leu, L; *N*-methylated Leu, mL) and relative production of all RXPs produced in both systems. **b**, The diagram shows the relative amounts (%) and peptide length (in numbers of amino acids) of fully methylated RXPs (solid line) produced in all systems.

a

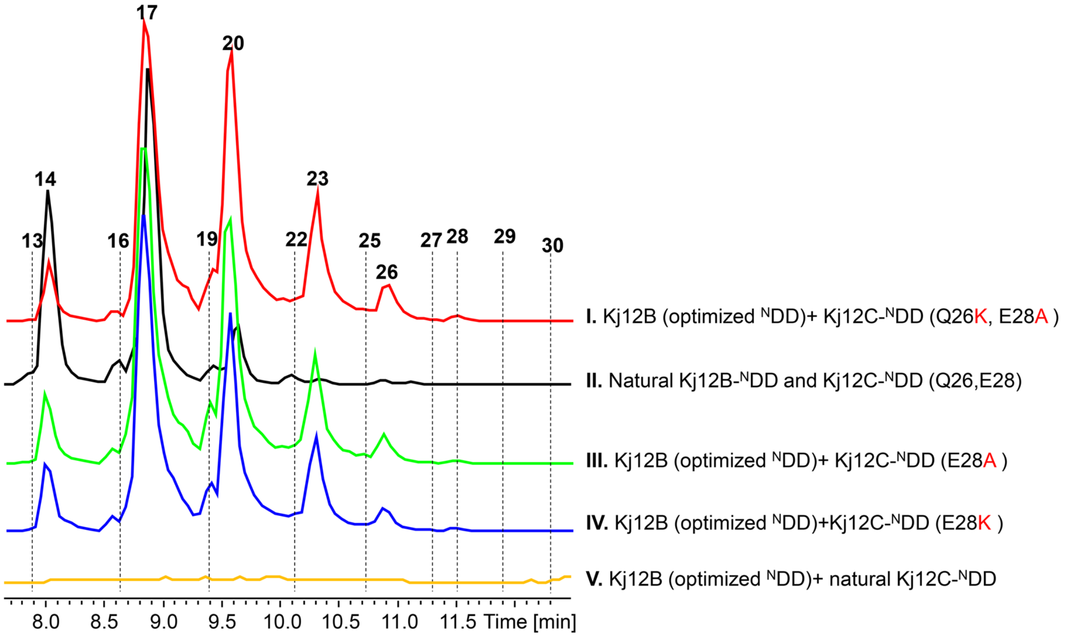

Compound	Rt/min	*m/z*	Structure	Relative RXP amount			
				I	II	III	IV
**13**	7.9	489.4	mL-L-mL-PEA	0.4 ± 0.2	4.3 ± 1.3	0.6 ± 0.2	0.6 ± 0.0
**14**	8	503.4	mL-mL-mL-PEA	11.1 ± 0.7	53.6 ± 6.7	14.5 ± 0.3	15.3 ± 1.1
**16**	8.6	616.5	mL-L-mL-mL-PEA	6.5 ± 2.2	11.7 ± 4.0	8.2 ± 8.2	7.6 ± 6.5
**17**	8.8	630.1	mL-mL-mL-mL-PEA	100.0 ± 10.0	100.0 ± 10.0	100.0 ± 10.0	100.0 ± 10.0
**19**	9.4	743.5	mL-L-mL-mL-mL-PEA	13.9 ± 4.1	7.7 ± 2.8	15.0 ± 9.6	13.7 ± 0.9
**20**	9.6	757.5	mL-mL-mL-mL-mL-PEA	75.6 ± 6.0	18.6 ± 10.0	58.8 ± 4.5	58.5 ± 3.6
**22**	10.2	870.4	mL-L-mL-mL-mL-mL-PEA	7.5 ± 0.7	1.5 ± 1.5	5.2 ± 2.3	9.0 ± 0.8
**23**	10.3	884.7	mL-mL-mL-mL-mL-mL-PEA	33.1 ± 14.4	2.1 ± 1.6	28.2 ± 7.5	25.5 ± 1.7
**25**	10.7	997.7	mL-L-mL-mL-mL-mL-mL-PEA	3.6 ± 1.8	0.06 ± 0.1	2.2 ± 9.0	2.0 ± 0.1
**26**	10.9	1011.6	mL-mL-mL-mL-mL-mL-mL-PEA	10.1 ± 2.2	0.11 ± 0.1	3.0 ± 3.1	1.4 ± 0.6
**27**	11.3	1124.8	mL-L-mL-mL-mL-mL-mL-mL-PEA	0.1 ± 0.0	–	0.2 ± 0.3	0.1 ± 0.1
**28**	11.5	1138.8	mL-mL-mL-mL-mL-mL-mL-mL-PEA	1.6 ± 0.6	–	1.2 ± 0.1	0.9 ± 0.2
**29**	11.9	1265.9	mL-mL-mL-mL-mL-mL-mL-mL-mL-PEA	0.1 ± 0.0	–	0.1 ± 0.1	0.1 ± 0.01
**30**	12.3	1392.7	mL-mL-mL-mL-mL-mL-mL-mL-mL-mL-PEA	0.014 ± 0.008	–	0.010 ± 0.012	0.009 ± 0.006

b

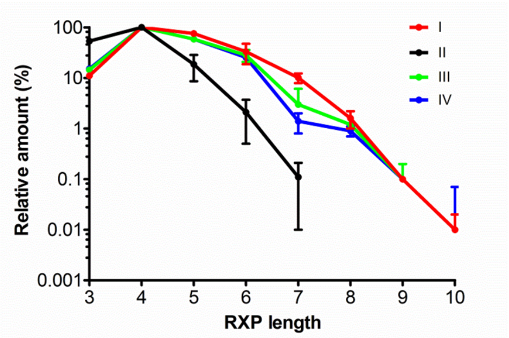

Supplementary Figure 24. Comparison of RXPs produced in artificial Kj12BC. a, Val specific A-MT from Kj12B was replaced against that from VietB for Leu specificity) with natural Kj12B-^{N}DD and Kj12C-^{N}DD (black, II) or optimized Kj12B-^{N}DD (K24R, K26Q, K28E) and Kj12C-^{N}DD in different modifications: (Q26K, E28A) (red, I); (E28A) (green, III); (E28K) (sapphire, IV) as well as optimized Kj12B-^{N}DD coexpressed with natural Kj12C-^{N}DD (yellow, V). The table shows the retention time (Rt), detected masses (*m/z*) in $[M+H]^{+}$, structures (simplified codes, Leu, L; *N*-methylated Leu, mL) and relative production of all RXPs produced in both systems. **b**, The diagram shows the relative amounts (%) and peptide length (in numbers of amino acid composition) of fully methylated RXPs (solid line) produced in all systems.

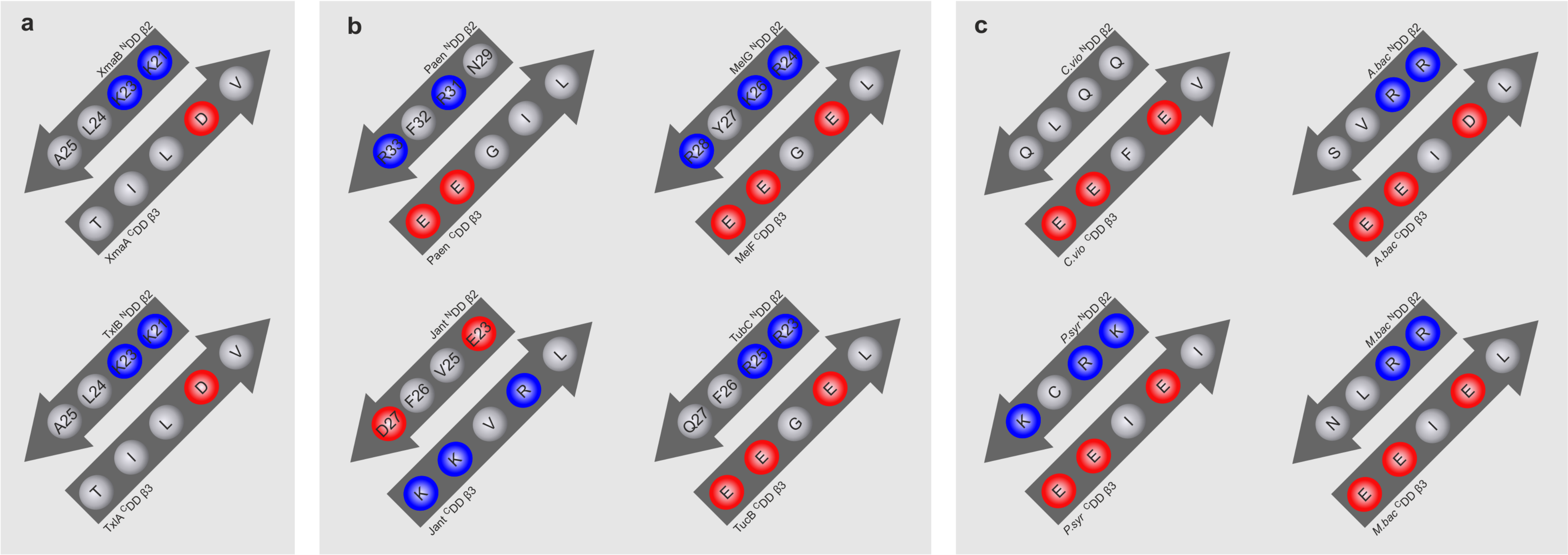

Supplementary Figure 25. Schematic representation of β-sheets with key residues mediating DD interaction in docking domains of the same structural class as Kj12BC. a, Two examples are shown from Supplementary Figure 2b, the interaction of this group is always based on a salt bridge between a positive residue on the ^{N}DD and a negative residue on the ^{C}DD and a hydrophobic interaction. **b**, Four examples are shown from Supplementary Figure 2c; this group of DD contains different types of key residues combinations; Paen ^{N}DD-^{C}DD interaction consists of a hydrophobic interaction and salt bridge between R33 of the ^{N}DD and two negative residues on the ^{C}DD, the Jant ^{N}DD-^{C}DD interaction consists of two salt bridges with negative residues on the ^{N}DD an positive residues on the ^{C}DD, MelG-^{N}DD MelF-^{C}DD interaction also consists of two salt bridges with positive residues on the ^{N}DD an negative residues on the ^{C}DD. In the case of TubC ^{N}DD- TubB ^{C}DD interaction, a salt bridge is replaced by a hydrogen bond between a Q and an E. Positive residues are shown as blue circles, negative residues as red circles and hydrophobic residues as white circles. **c**, *C.vio: Chromobacterium violaceum* strain 968, *P.syr: Pseudomonas syringae pv. syringae* B728a, *A.bac: Acidobacteria bacterium* SCN 69-37, *M.bac: Mycolicibacterium bacteremicum.*

Supplementary Tables

Supplementary Table 1. Structural statistics for the NMR solution structures of Kj12A-^{N}DD, Kj12B-^{N}DD, Kj12C-^{N}DD and Kj12C-^{N}DD-Kj12B-^{C}DD.

	**Kj12A-^{N}DD**	**Kj12B-^{N}DD**	**Kj12C-^{N}DD**	**Kj12C-^{N}DD-Kj12B-^{C}DD**
**Conformational restricting restraints**				
Total NOE distance restraints	1577	1783	1559	2370
intraresidue |i = j|	343	373	352	466
sequential |i - j| = 1	403	443	382	616
medium-range 1 <|i - j| < 5	498	486	391	653
long-range |i - j| ⩾ 5	333	481	434	635
Dihedral angle restraints (Talos+)	102	96	106	140
No. of restraint per residue	27.1	30.3	25.7	25.8
No. of long-range restraints per residue	5.4	7.8	6.8	6.9
**Residual restraint violations[a]**				
Average no. of distance violations per structure				
0.1-0.2 Å	4.5	4.35	2.35	9.95
0.2-0.5 Å	0.15	0.3	0.2	08
>0.5 Å	0	0	0	0.25
Average no. of dihedral angle violations per structure				
1-10°	13.5	3.75	7.55	15.2
>10°	0	0	0	2.4
**Model quality (ordered residues)[a]**				
RMSD backbone atoms (Å)	0.1	0.2	0.1	0.2
RMSD heavy atoms (Å)	0.5	0.6	0.5	0.5
RMSD bond lengths (Å)	0.001	0.001	0.001	0.001
RMSD bond angles (°)	0.2	0.2	0.2	0.2
**MolProbity Ramachandran statistics[a]**				
Most favored regions	97.3 %	96.7 %	92.5 %	93.2 %
Allowed regions	2.7 %	3.3 %	7.5 %	5.1 %
Disallowed regions	0 %	0.0 %	0.1 %	1.7 %
**Global quality scores (raw/Z score)[a]**				
Verify3D	0.38/-1.28	0.41/-0.8	0.33/-2.09	0.31/-2.41
Prosall	0.94/1.20	1.09/1.82	1.03/1.57	
PROCHECK (φ-ψ)	0.07/-0.58	0.04/0.47	-0.17/-0.35	-0.09/-0.04
PROCHECK (all)	-0.55/-3.25	-0.53/-3.13	-0.55/-3.25	-0.54/-3.19
MolProbity clash score	30.80/-3.76	27.64/-3.22	29.68/-3.57	13.64/-0.82
**Model contents**				
Ordered residue ranges (HetNOE > 0.6)	2-58	3-58	3-57	3-58,1564-1568
Total no. of residues	63	62	63	99
BMRB accession number				
PDB ID code	6EWS	6EWT	6EWU	6EWV

[a] calculated using PSVS 1.5 for using ordered residues (HetNOE > 0.6) (Bhattacharya et al., 2007). Average distance violations were calculated using the sum over r-6.

Supplementary Table 2. ITC titration experiments of all RXP ^{N}DDs with ^{C}DDs.

			K_D [μM]	N [sites]	ΔH [kcal/mol]	ΔS [cal/mol/deg]	c-values
**Kj12A-^{N}DD**		**Kj12A-^{C}DD**	weak				
		**Kj12B-^{C}DD**	8±4	0.81±0.01	-4±2	9±7	12±6
**Kj12B-^{N}DD**		**Kj12A-^{C}DD**	weak				
		**Kj12B-^{C}DD**	62±8	1±0	-5.4±0.6	0.8±1.8	1.6±0.2
		**Kj12B-^{C}DD$_{short}$ R1565E**	13±2	1.15±0.08	3.31±0.06	11.2±0.5	9.1±0.6
**Kj12B-^{N}DD**	**K28E**	**Kj12B-^{C}DD$_{short}$**	33±8	1±0	-5±5	3±2	3.1±0.7
	**K26Q, K28E**	**Kj12B-^{C}DD$_{short}$**	20.4±0.4	1±0	-6±3	2±2	4.89±0.09
	**K24R, K26Q, K28E**	**Kj12B-^{C}DD$_{short}$**	11.8±0.9	0.97±0.02	-2.8±0.1	12.7±0.1	8.2±0.8
**Kj12C-^{N}DD**		**Kj12A-^{C}DD**	weak				
		**Kj12A-^{C}DD$_{short}$ E1169R, H1171E**	3.5±0.1	1.3±0.2	-6.3±0.9	1.7±0.5	38±4
		**Kj12B-^{C}DD**	8±6	0.90±0.12	-5.0±3.0	6±13	15±12
		**Kj12B-^{C}DD$_{short}$**	15±3	1.05±0.11	-5.3±0.1	3.9±0.2	7.2±0.5
		**Kj12B-^{C}DD$_{short}$ L1564A**	weak				
		**R1565E**	weak				
		**G1566A**	weak				
		**E1567R**	64±7	1.08±0.05	-4.5±0.2	3.8±0.9	1.7±0.1
		**E1567A**	24±6	0.90±0.15	-18±2	11±1	3.9±0.3
		**I1568A**	very weak				
**Kj12C-NDD Q26K, E28A**		**Kj12B-^{C}DD$_{short}$**	80±8	1±0	-6.3±0.3	3±1	1.3±0.1
**Kj12C-^{N}DD-Cterm**		**Kj12B-^{C}DD$_{short}$**	8±6	1±0	-1±1	19±7	16±12
**Kj12C-Cterm**		**Kj12B-^{C}DD$_{short}$**	very weak				
**Kj12B-Tdom-^{C}DD**		**Kj12C-^{N}DD**	19±6	1.2±0.7	-2±1	17±3	6±2

Supplementary Table 3. Identified RXP-like DD pairs in other bacteria. In the β-sheets that mediate the DD interaction acidic and basic amino acids are shown in red and blue, respectively.

	GenBank accession No	^{C}DD	^{N}DD	^{C}DD final β-sheet*	^{N}DD β2-sheet*	^{C}DD	^{N}DD
Chromobacterium violaceum strain 968	EF210776.1	ABP57747.1 (DepC)	ABP57748.1 (DepD)	EEITL	QLQVQ	PKS-ACP-^{C}DD	^{N}DD-C-NRPS
Pseudomonas sp. 250J	GCA_001259595.1	WP_050705533.1	WP_050705534.1	EEFEV	KLRCK	T-OxRed-^{C}DD	^{N}DD-C-NRPS
Pseudomonas fluorescens	GCA_000012445.1	WP_011333692.1	WP_011333693.1	EEFEV	RLRCK	T-OxRed-^{C}DD	^{N}DD-C-NRPS
Pseudomonas moraviensis R28-S	GCA_000512275.1	WP_065617345.1	WP_065617344.1	EEFEV	RLRCK	T-OxRed-^{C}DD	^{N}DD-C-NRPS
Pseudomonas syringae pv. syringae B728a	GCA_000012245.1	WP_011267241.1	WP_011267242.1	EEIEI	KLRCK	T-OxRed-^{C}DD	^{N}DD-C-NRPS
Pseudomonas *coronafaciens* pv. *garcae*	LJQK01000091.1	WP_055004516.1	WP_055004515.1	EEIEI	KLRCK	T-OxRed-^{C}DD	^{N}DD-C-NRPS
Chondromyces crocatus Cm c5	GCA_001189295.1	WP_082362833.1	WP_050433124.1	EEGEI	KLRFR	PKS-ACP-^{C}DD	^{N}DD-C-NRPS-PKS
Angiococcus disciformis	AJ620477.1	CAF05648.1	CAF05649.1	EEGEL	RLRFQ	NRPS-T-^{C}DD	^{N}DD-C-NRPS
Cystobacter ferrugineus	GCA_001887355.1	WP_071905046.1	WP_071905047.1	EEGEI	RLRFQ	NRPS-T-^{C}DD	^{N}DD-C-NRPS
Melittangium lichenicola	AJ557546.1	CAD89777.1	CAD89778.1	EEGEL	RLKYR	PKS-ACP-^{C}DD	^{N}DD-C-NRPS
Cystobacter fuscus strain DSM 52655	GCA_002305875.1	WP_095988360.1	WP_095988359.1	EELTL	SLRLR	PKS-ACP-^{C}DD	^{N}DD-C-NRPS
Stigmatella aurantiaca DW4/3-1	GCA_000165485.1	ADO71804.1	ADO71805.1	EEGEL	RLKYR	PKS-ACP-^{C}DD	^{N}DD-C-NRPS
Acidobacteria bacterium SCN 69-37	GCA_001724025.1	ODS52927.1	ODS52926.1	EEIDL	RLRVS	PKS-ACP-^{C}DD	^{N}DD-C-NRPS
Mycolicibacterium bacteremicum	GCA_002086115.1	ORA04543.1	ORA04542.1	EEIEL	RLRLN	NRPS-T-^{C}DD	^{N}DD-TE
Herpetosiphon aurantiacus DSM 785	GCA_000018565.1	ABX04503.1	ABX04504.1	EEIEL	NLRVN	PKS-ACP-^{C}DD	^{N}DD-C-NRPS
Microcystis aeruginosa K-139	AB481215.1	BAH22764.1	BAH22765.1	EDGEL	KLRFQ	NRPS-T-^{C}DD	^{N}DD-C-NRPS
Microcystis sp. NIVA-CYA 172/5	DQ075244.1	AAZ03552.1	AAZ03553.1	EEGEL	KLRYR	NRPS-T-^{C}DD	^{N}DD-DH stand alone
Nostoc sp. CENA543	GCA_002896875.1	WP_103137408.1	WP_103137407.1	EEDYL	QLRYR	(A?-KR)-ACP-^{C}DD	^{N}DD-C-NRPS
Planktothrix agardhii NIES-205	EU109504.1	ABW84365.1	ABW84366.1	EEGEL	KLRYQ	NRPS-T-^{C}DD	^{N}DD-C-NRPS
Duganella sacchari	GCA_900143065.1	SHM78877.1	SHM78841.1	TTIRI	ELVLE	NRPS-T-^{C}DD	^{N}DD-C-NRPS
Janthinobacterium sp. HH01	GCA_000335815.1	WP_008447581.1	WP_008447582.1	KRVRI	ELVLD	NRPS-T-^{C}DD	^{N}DD-C-NRPS
Paenibacillus elgii B69	GCA_900188505.1	WP_088834641.1	WP_088834640.1	EGVLE	KLRFR	T-OxRed-^{C}DD	^{N}DD-C-NRPS-PKS
Paenibacillus larvae subsp. *pulvifaciens*	GCA_002082155.1	WP_083039736.1	WP_083039734.1	EEGIL	NLRFR	T-OxRed-^{C}DD	^{N}DD-C-NRPS
Paenibacillus tyrfis	GCA_000722545.1	WP_051775011.1	WP_051775010.1	EGVLE	KLRFR	T-OxRed-^{C}DD	^{N}DD-C-NRPS-PKS

*the β-sheet position refers to the Supplementary Figure 2 nomenclature in contrast to the covalently linked NMR structure position designation.

Supplementary Table 4. HR-MS data of synthesized short peptides.

peptide	sum formula	calc $[M+2H]^{2+}$	found $[M+2H]^{2+}$	Δ ppm
QEYLRGEA	$C_{41}H_{64}N_{12}O_{15}$	483.2380	483.2382	-0.5
QEYLRGEI	$C_{44}H_{70}N_{12}O_{15}$	504.2615	504.2619	-0.9
QEYLRGRI	$C_{45}H_{75}N_{15}O_{13}$	517.7907	517.7905	0.4
QEYLEGEI	$C_{43}H_{65}N_{9}O_{17}$	490.7322	490.7319	0.5
QEYLRAEI	$C_{45}H_{72}N_{12}O_{15}$	511.2693	511.2698	-0.9
QEYARGEI	$C_{41}H_{64}N_{12}O_{15}$	483.2380	483.2382	-0.5
QEYLRGAI	$C_{42}H_{68}N_{12}O_{13}$	475.2587	475.2587	0.1
YIHLLKEKRKHFQA	$C_{85}H_{135}N_{25}O_{19}$	906.0256	906.0247	0.9
YQKLLRRGEI	$C_{51}H_{86}N_{14}O_{14}$	560.3297	560.3291	1.0
QKLLRGEI	$C_{42}H_{77}N_{13}O_{12}$	478.7980	478.7981	-0.2

Supplementary Table 5. Bacterial strains used in this study.

Strain	Relevant Genotype	Reference/Strain No.
E.coli		
DH10BMtaA	F– *mcr*A, Δ(*mrr-hsd*RMS-*mcr*BC), Φ80*lac*ZΔM15, Δ*lac*X74, *rec*A1, *end*A1, *ara*D139, Δ(*ara leu*)7697, *gal*U, *gal*K, *rps*L, *nup*G, λ–, *entD::mtaA*	[1, 4]
BL21 GOLD (DE)	*E. coli* B F^- *ompT hsdS*(r_B^- m_B^-) dcm^+ Tet^r *gal* λ(DE3) *endA* Hte	Agilent
Xenorhabdus stockiae	Wild type	KJ12.1

Supplementary Table 6. Primers used in this study.

Primer	Sequence (5'-3')	Targeting DNA fragment	Plasmid
hSUMO_fw	TTAAGAAGGAGATATACATATGGCTAGCGGTCATCCATC	pET11a-mod-His6 SUMO-NisI97-226	
hSUMO_rev	CCACCAATCTGTTCACGATG		pET11a-modified
pET11a-for	TAAGGATCCGGCTGCTAAC	pET11a (pCK_0415)	
pET11a-rev	ATGTATATCTCCTTCTTAAAG		
ck0011	CCATCGTGAACAGATTGGTGGTATGATAGATGCAGCGCAGAT	*Kj12C-^{N}DD* from *X. stockiae* KJ12.1 (192 bp)	
ck0010	CTTTGTTAGCAGCCGGATCCTTATTTAGTTTGTTCTTCTGAATCAAGCT	pET11a (mod) vector backbone (5,935 bp)	pCK_0500
pET11a-for	TAAGGATCCGGCTGCTAAC		
pET11a-smt3-rev	ATGTATATCTCCTTCTTAAAGTTAAACAAAATTATTTCTA		
ck0058	CATCGTGAACAGATTGGTGGTATGAAGAATGCCGCTCAA	*Kj12C-^{N}DD* from *X. stockiae* KJ12.1 (192 bp)	
ck0059	TTTGTTAGCAGCCGGATCCTTATTGTGTTTCTTCTTTTGCATCG	pET11a (mod) vector backbone (5,935 bp)	pCK_0533
pET11a-for	TAAGGATCCGGCTGCTAAC		
pET11a-smt3-rev	ATGTATATCTCCTTCTTAAAGTTAAACAAAATTATTTCTA		
ck0060	CATCGTGAACAGATTGGTGGTATGAAAAATGCAGCTAAGATTGTG	*Kj12B-^{N}DD* from *X. stockiae* KJ12.1 (192 bp)	
ck0061	TTTGTTAGCAGCCGGATCCTTACGATTCTTCTTCTGATTCAAGCT		pCK_0533
pET11a-for	TAAGGATCCGGCTGCTAAC	pET11a (mod) vector backbone (5,935 bp)	
pET11a-smt3-rev	ATGTATATCTCCTTCTTAAAGTTAAACAAAATTATTTCTA		
ck0100	GGATCGGGTTCGGGCAGTATGATAGATGCAGCGCAGA	pCK_0500 and subsequent ligation of the PCR product	pCK_0540
ck0101	GGAACCTGAACCACTACCACCACCAATCTGTTCACGA		
ck0102	CCATCGTGAACAGATTGGTGGTCTTCTCAAAGAAAAAAGAAAACATTTTC	pCK_0540, vector part fused to	
ck0103	ATCCGGAACCTGAACCACTACCTATTTCACCTCTTAAATATTCCTGAGATG		pCK_0541
ck0104	GAAATAGGTAGTGGTTCAGGTTCCG	*Kj12C-^{N}DD*	
ck0105	GAGAAGACCACCAATCTGTTCACGAT		
ck0109	GGATCGGGTTCGGGCAGTTAAGGATCCGGCTGCTAAC	pCK_0500 and subsequent ligation of the PCR product	pCK_0544
ck0110	GGAACCTGAACCACTACCTTTAGTTTGTTCTTCTGAATCAAGC		
ck0111	TTCCGGATCGGGTTCGGGCAGTCTTCTCAAAGAAAAAAGAAAACATTTTC	pCK0544, vector part fused to	
ck0113	GAAATATAAGGATCCGGCTGCTAAC		pCK_0545
ck0112	TTTGTTAGCAGCCGGATCCTTATATTTCACCTCTTAAATATTCCTGAGA	*Kj12C-^{C}DD*	
ck0115	CTTCTCAAAGAAAAAAGAAAACATTTTCAG		
ck0359	TATGAAACCAATCGTGATAGTATTCCATC	pCK_0534 as template, introducing a K28E codon change into Kj12B-^{N}DD from *X. stockiae* KJ12.1 (192 bp)	
ck0360	TTTTAGTTTATTATCGCTAACAAATAGAGTAATTCC		pCK_0630
ck0359	TATGAAACCAATCGTGATAGTATTCCATC	pCK_0534 as template, introducing K28E and K26Q codon changes into *Kj12B-^{N}DD* from *X. stockiae* KJ12.1 (192 bp)	
ck0361	TTGTAGTTTATTATCGCTAACAAATAGAGTAATTCC		pCK_0631
ck0359	TATGAAACCAATCGTGATAGTATTCCATC	pCK_0534 as template, introducing K28E, K26Q and K24R codon changes into *Kj12B-^{N}DD*	
ck0363	TTGTAGTCTATTATCGCTAACAAATAGAGTAATTCC		pCK_0632

		from *X. stockiae* KJ12.1 (192 bp)	
ck0364<br>ck0365	AATACGCAACCAGTCGTGACAGC<br>TTAACCGATTGTTAACAACAAATAGAGTAATCCC	pCK_0500 as template, introducing Q26K and E28A codon changes into *Kj12C-^{N}DD* from *X. stockiae* KJ12.1	pCK_0640
jw0075<br>jw0076<br>pET11a-for<br>pET11a-smt3-rev	CATCGTGAACAGATTGGTGGTAATGAAGATGATTTACGTCGGCAAACCTATG<br>TTTGTTAGCAGCCGGATCCTTATATTTCACCTCTTAAATATTCCTGAGATGAATTTTGC<br>TAAGGATCCGGCTGCTAAC<br>ATGTATATCTCCTTCTTAAAGTTAAACAAAATTATTTCTA	*Kj12B-Tdom-^{N}DD* from *X. stockiae* KJ12.1 (373 bp)	pJW58
SN_KJ12C_Ndd_Fw1<br>SN_KJ12C_Ndd_Rv1<br>SN_pCOLA_Ndd_Fw<br>SN_pCOLA_Ndd_Rv	TTAACTTTAATAAGGAGATATACCATGATAGATGCAGCGCAG<br>ATGTCCCTGGAAGTATAGGTTCTCTCCATAAAACTGGCTGATAC<br>GAGAACCTATACTTCCAGGGACATCACCATCATCACCACTAATGCTTAAGTCGAACAG<br>GGTATATCTCCTTATTAAAGTTAAAC	*Kj12C-^{N}DD-Cterm* from *X. stockiae* KJ12.1 (192 bp)<br>pCOLA_Duet	pCOLA-Kj12C-^{N}DD-Cterm
SN_KJ12C_w/o_Fw (5' Phos)<br>SN_KJ12C_w/o_Rv	AATGGAAAAGTGCCTATTTC<br>CATGGTATATCTCCTTATTAAAGTTAAAC	*pCOLA-Kj12C-^{N}DD-Cterm*	pCOLA-Kj12C-Cterm
XC3-Fw<br>XC204-Rv	ATGAAGAATGCCGCTCAAATTGTGGATG<br>ACAATCTTAGCTGCATTTTTCATATATGACCTTCCAATAG	*kj12A* from *X. stockiae* KJ12.1 (3,538 bp)	
XC204-Fw<br>XC3-Rv	AGGTCATATATGAAAAATGCAGCTAAGATTGTG<br>TTATCCATAAAACTGGCTGATACTCTC	The region encoding KJ12BC (A-MT:VietB) from *X. stockiae* KJ12.1 (6,275 bp)	pCX178
XC4-Fw<br>XC4-Rv	AAGAGAGTATCAGCCAGTTTTATGGATAACAATTAATCATCGGCTCGTA<br>AGCCTCATCCACAATTTGAGCGGCATTCTTCATGGAATTCCTCCTGTTAGCCC	pCOLA-ara-tacI vector backbone (3,368 bp)	
XC3-Fw<br>XC280-Rv	ATGAAGAATGCCGCTCAAATTGTGGATG<br>AGCTGCATTTTTCATATTTCACCTCTCAATAG	*kj12A* with double point mutation on ^{C}DD (E1169R and H1171E) from *X. stockiae* KJ12.1 (3,530 bp)	
XC280-Fw<br>XC3-Rv	TGAGAGGTGAAATATGAAAAATGCAGCTAAG<br>TTATCCATAAAACTGGCTGATACTCTC	*kj12BC* from *X. stockiae KJ12.1* (6,305 bp)	pCX242
XC4-Fw<br>XC4-Rv	AAGAGAGTATCAGCCAGTTTTATGGATAACAATTAATCATCGGCTCGTA<br>AGCCTCATCCACAATTTGAGCGGCATTCTTCATGGAATTCCTCCTGTTAGCCC	pCOLA-ara-tacI vector backbone (3,368 bp)	
XC3-Fw<br>XC282-Rv	ATGAAGAATGCCGCTCAAATTGTGGATG<br>AGCTTCGTTCACAATCTTAGC	DNA fragment encoding Kj12A-^{C}DD with double point mutations kj12A(E1169R and H1171E) from *X. stockiae* KJ12.1 (3,519 bp)	pCX244
XC282-Fw<br>XC4-Rv	AGGTGAAATATGACAATTAATCATCGGCTCG<br>AGCTTCGTTCACAATCTTAGCTGCATTTTTCATGGAATTCCTCCTGTTAGCC	pCOLA-ara-tacI vector backbone (3,368 bp)	
XC3-Fw<br>XC280-Rv	ATGAAGAATGCCGCTCAAATTGTGGATG<br>AGCTGCATTTTTCATATTTCACCTCTCAATAG	*kj12A* with double point mutation on ^{C}DD (E1169R and H1171E) from *X. stockiae* KJ12.1 (3,530 bp)	

XC280-Fw<br>XC3-Rv	TGAGAGGTGAAATATGAAAAATGCAGCTAAG<br>TTATCCATAAAACTGGCTGATACTCTC	*kj12BC (A-MT:vietB)* from pCX74 (6,305 bp)	pCX253
XC4-Fw<br>XC4-Rv	AAGAGAGTATCAGCCAGTTTTATGGATAACAATTAATCATCGGCTCGTA<br>AGCCTCATCCACAATTTGAGCGGCATTCTTCATGGAATTCCTCCTGTTAGCCC	pCOLA-ara-tacI vector backbone (3,368 bp)	
XC295-Fw<br>XC30-Rv	AGACTAAAATATGAAACCAATCGTGATAGTATTCCATC<br>TCATATTTCACCTCTTAAATATTCCTG	DNA fragment encoding Kj12B (A-MT:VietB) with double point mutations (K24R and K28E) on ^{N}DD from pCX88 (4,637 bp)	pCX256
XC31-Fw<br>XC295-Rv	ATCTCAGGAATATTTAAGAGGTGAAATATGACAATTAATCATCGGCTCGTATAATG<br>ACTATCACGATTGGTTTCATATTTTAGTCTATTATCGCTAACAAATAGAG	pCX16 vector backbone (3,212 bp)	
XC296-Fw<br>XC30-Rv	ATGAAACCAATCGTGATAGTATTC<br>TCATATTTCACCTCTTAAATATTCCTG	DNA fragment encoding Kj12B (A-MT:VietB) with single point mutation (K28E) on ^{N}DD from pCX88 (4,637 bp)	pCX257
XC31-Fw<br>XC296-Rv	ATCTCAGGAATATTTAAGAGGTGAAATATGACAATTAATCATCGGCTCGTATAATG<br>ATGGAATACTATCACGATTGGTTTCATATTTTAGTTTATTATCGCTAAC	pCX16 vector backbone (3,212 bp)	
XC297-Fw<br>XC30-Rv	AGACTACAATATGAAACCAATCGTGATAGTATTCCATC<br>TCATATTTCACCTCTTAAATATTCCTG	DNA fragment encoding Kj12B (A-MT:VietB) with triple point mutations (K24R, K26Q and K28E) on ^{N}DD from pCX88 (4,637 bp)	pCX258
XC31-Fw<br>XC297-Rv	ATCTCAGGAATATTTAAGAGGTGAAATATGACAATTAATCATCGGCTCGTATAATG<br>ACTATCACGATTGGTTTCATATTGTAGTCTATTATCGCTAACAAATAGAG	pCX16 vector backbone (3,212 bp)	
XC300-Fw<br>XC30-Rv	ACTACAATATGAAACCAATCGTGATAGTATTCCATC<br>TCATATTTCACCTCTTAAATATTCCTG	DNA fragment encoding Kj12B (A-MT:VietB) with double point mutations (K26Q and K28E) on ^{N}DD from pCX88 (4,637 bp)	pCX261
XC31-Fw<br>XC300-Rv	ATCTCAGGAATATTTAAGAGGTGAAATATGACAATTAATCATCGGCTCGTATAATG<br>ACTATCACGATTGGTTTCATATTGTAGTTTATTATCGCTAACAAATAGAG	pCX16 vector backbone (3,212 bp)	
XC301-Fw<br>XC30-Rv	ACTACAATATAAAACCAATCGTGATAGTATTCCATC<br>TCATATTTCACCTCTTAAATATTCCTG	DNA fragment encoding Kj12B (A-MT:VietB) with single point mutation (K26Q) on ^{N}DD from pCX88 (4,637 bp)	pCX262
XC31-Fw<br>XC301-Rv	ATCTCAGGAATATTTAAGAGGTGAAATATGACAATTAATCATCGGCTCGTATAATG<br>ACTATCACGATTGGTTTTATATTGTAGTTTATTATCGCTAACAAATAGAG	pCX16 vector backbone (3,212 bp)	
XC304-Fw<br>XC3-Rv	ACAATACAAAACCAGTCGTGACAGCATTCC<br>TTATCCATAAAACTGGCTGATACTCTC	DNA fragment encoding Kj12C with single point mutation (E28K) on ^{N}DD from pCX19 (1,510 bp)	pCX267

XC4-Fw<br>XC304-Rv	AAGAGAGTATCAGCCAGTTTTATGGATAACAATTAATCATCGGCTCGTA<br>ATGCTGTCACGACTGGTTTTGTATTGTAACCGATTGTTAAC	pCDF-ara-tacI vector backbone (3,466 bp)	
XC305-Fw<br>XC3-Rv	ACAATACGCAACCAGTCGTGACAGCATTCC<br>TTATCCATAAAACTGGCTGATACTCTC	DNA fragment encoding Kj12C with single point mutation (E28A) on ^{N}DD from pCX19 (1,510 bp)	pCX268
XC4-Fw<br>XC305-Rv	AAGAGAGTATCAGCCAGTTTTATGGATAACAATTAATCATCGGCTCGTA<br>ATGCTGTCACGACTGGTTGCGTATTGTAACCGATTGTTAAC	pCDF-ara-tacI vector backbone (3,466 bp)	
XC306-Fw<br>XC3-Rv	ATCGGTTAAAATACAAAACCAGTCGTGACAGCATTCC<br>TTATCCATAAAACTGGCTGATACTCTC	DNA fragment encoding Kj12C with double point mutations (Q26E and E28A) on ^{N}DD from pCX19 (1,510 bp)	pCX269
XC4-Fw<br>XC306-Rv	AAGAGAGTATCAGCCAGTTTTATGGATAACAATTAATCATCGGCTCGTA<br>TGTCACGACTGGTTTTGTATTTTAACCGATTGTTAACAACAAATAG	pCDF-ara-tacI vector backbone (3,466 bp)	

Supplementary Table 7. Plasmids used in this study.

Plasmid	Description	References
pET11a modified	5,938 bp, modified from pET11a, the operon under the control of T7 promoter was modified by introduction of N-terminal His × 6-smt3 tag, His x 6-smt3 sequence from a plasmid used in another study (Hacker, Christ 2015) (Am^R)	This study
pCK_0500	6,127 bp, vector, the Kj12C-^{N}DD was fused N-terminally to *smt3* into pET11a-modified, under control of T7 promoter (Am^R)	This study
pCK_0533	6,127 bp, vector, Kj12A-^{N}DD was fused N-terminally to *smt3* into pET11a-modified, under control of T7 promoter (Am^R)	This study
pCK_0534	6,124 bp, vector fusing the Kj12B-^{N}DD N-terminally to *smt3* into pET11a-modified, under control of T7 promoter (Am^R)	This study
pCK_0540	6,163 bp, vector, adding 12 GS-codons in frame N-terminally to *smt3* and C-terminally to the Kj12C-^{N}DD into pCK_0500, under control of T7 promoter (Am^R)	This study
pCK_0541	6,235 bp, vector, the Kj12B-^{C}DD was fused in frame C-terminally to *smt3* and N-terminally to Kj12C-^{N}DD originating from vector vector pCK_0540, under control of T7 promoter (Am^R)	This study
pCK_0544	6,163 bp, vector, adding 12 GS-codons in frame C-terminally to Kj12C-^{N}DD from pCK_0500, under control of T7 promoter (Am^R)	This study
pCK_0545	6,235 bp, vector, the Kj12B-^{C}DD was fused in frame N-terminally to the 12-GS-linker form pCK_0544, under control of T7 promoter (Am^R)	This study
pCK_0630	6,124 bp, vector based on pCK_0534, one codon change was introduced into the Kj12B-^{N}DD domain (K28E in respect to the Kj12B-^{N}DD)	This study
pCK_0631	6,124 bp, vector based on pCK_0534, two codon changes were introduced into the Kj12B-^{N}DD domain (K26Q and K28E, in respect to the Kj12B-^{N}DD).	This study
pCK_0632	6,124 bp, vector based on pCK_0534, three codon changes were introduced into the Kj12B-^{N}DD domain (K24R, K26Q and K28E, in respect to the Kj12B-^{N}DD).	This study
pCK_0640	6,127 bp, vector based on pCK_500, two codon changes were introduced resulting into the Kj12C-^{N}DD domain (Q26K and E28A, in respect to the Kj12C-^{N}DD).	This study
pJW58	6,268 bp, vector, Kj12B-Tdom-^{C}DD was fused N-terminally to *smt3* into pET11a-modified, under control of T7 promoter (Am^R)	This study
pKj12C-Cterm-^{N}DD	5252 pb, pCOLA vector, Kj12C-Cterm-^{N}DD was fused C-terminal with a His_6-tag and a TEV cleavage site, under control of a T7 promotor (Km^R)	This study
pKj12C-Cterm-^{N}DD	5015 pb, pCOLA vector, Kj12C-Cterm was fused C-terminal with a His_6-tag and a TEV cleavage site, under control of a T7 promotor (Km^R)	This study
pCX16	3,189 bp, modified from pCOLA-ara-tacI with Km^R replaced by chloramphenicol resistance gene (Cm^R)	[1]
pCX88	4,707 bp, *kj12B* from *X. stokiae* KJ12.1 with A-MT gene replaced by the A-MT encoding gene from *vietB* in *X.vietnamensis* DSM 22392, assembled into pCX16 (Cm^R)	[1]
pCX74	6,287 bp, *kj12BC* from *X. stokiae* KJ12.1 with A-MT gene replaced by the A-MT encoding gene from *vietB* in *X. vietnamensis* DSM 22392, assembled into pCX16 (Cm^R)	[1]
pCX26	7,896 bp, *kj12B* from *X. stockiae* KJ12.1, assembled into pCX16, (Cm^R)	[1]

Plasmid	Description	References
pCX19	5,005 bp, *kj12C* from *X. stockiae* KJ12.1 assembled into pCDF-ara-tacI, (Sm^R)	[1]
pCX3	16,107 bp, kj12 gene cluster from *X. stockiae* KJ12.1 genomic DNA assembled into pCOLA-ara-tacI, Km^R	[1]
pCX178	13,032 bp, DNA fragment encoding Kj12ABC with A-MT domain replaced by that of VietB from *X. vientnamensis* for Leu specificity (A-MT:VietB), assembled into pCOLA-ara-tacI (Km^R)	This study
pCX242	13,032 bp, DNA fragment encoding Kj12ABC from *X. stockiae* KJ12.1 with double point mutations on Kj12A-^{C}DD (E1169R and H1171E), assembled into pCOLA-ara-tacI (Km^R)	This study
pCX244	6,825 bp, DNA fragment encoding Kj12A from *X. stockiae* KJ12.1 with point mutation on Kj12A-^{C}DD (E1169R and H1171E), assembled into pCOLA-ara-tacI (Km^R)	This study
pCX253	13,032 bp, DNA fragment encoding Kj12ABC with A-MT domain exchanged against that of VietB from *X. vientnamensis* for Leu specificity (A-MT:VietB) and point mutation on Kj12A-^{C}DD (E1169R and H1171E), assembled into pCOLA-ara-tacI (Km^R)	This study
pCX256	7,857 bp, DNA fragment encoding Kj12B (A-MT:VietB) with double point mutations on Kj12B-^{N}DD (K28E and K24R), assembled into pCX16 (Cm^R)	This study
pCX257	7,857 bp, DNA fragment encoding Kj12B (A-MT:VietB) with single point mutation on Kj12B-^{N}DD (K28E), assembled into pCX16 (Cm^R)	This study
pCX258	7,857 bp, DNA fragment encoding Kj12B (A-MT:VietB) with triple point mutations on Kj12B-^{N}DD (K28E, K24R and K26Q), assembled into pCX16 (Cm^R)	This study
pCX261	7,857 bp, DNA fragment encoding Kj12B (A-MT:VietB) with double point mutations on Kj12B-^{N}DD (K26Q and K28E), assembled into pCX16 (Cm^R)	This study
pCX262	7,857 bp, DNA fragment encoding Kj12B (A-MT:VietB) with single point mutation on Kj12B-^{N}DD (K26Q), assembled into pCX16 (Cm^R)	This study
pCX267	4,949 bp, DNA fragment encoding Kj12C from *X. stockiae* KJ12.1 with single point mutation on Kj12C-^{N}DD (E28K), assembled into pCDF-ara-tacI (Sm^R)	This study
pCX268	4,949 bp, DNA fragment encoding Kj12C from *X. stockiae* KJ12.1 with single point mutation on Kj12C-^{N}DD (E28A), assembled into pCDF-ara-tacI (Sm^R)	This study
pCX269	4,949 bp, DNA fragment encoding Kj12C from *X. stockiae* KJ12.1 with double point mutations on Kj12C-^{N}DD (Q26K and E28A), assembled into pCDF-ara-tacI (Sm^R)	This study

Am^R: ampicilin resistance; Km^R : kanamycin resistance; Cm^R: chloramphenicol resistance; Sm^R: spectinomycin resistance

Supplementary References

1. Cai, X. *et al.* Entomopathogenic bacteria use multiple mechanisms for bioactive peptide library design. *Nat Chem* **9,** 379–386 (2017).
2. Tobias, N. J. *et al.* Natural product diversity associated with the nematode symbionts *Photorhabdus* and *Xenorhabdus*. *Nature Microbiology* **2,** 1676–1685 (2017).
3. Kronenwerth, M. *et al.* Characterisation of taxlllaids A-G; natural products from *Xenorhabdus indica*. *Chem. Eur. J.* **20,** 17478–17487 (2014).
4. Edgar, R. C. MUSCLE: a multiple sequence alignment method with reduced time and space complexity. *BMC Bioinformatics* **5,** 113 (2004).
5. Edgar, R. C. MUSCLE: multiple sequence alignment with high accuracy and high throughput. *Nucleic Acids Res.* **32,** 1792–1797 (2004).
6. Hacker, C. *et al.* The Solution Structure of the Lantibiotic Immunity Protein NisI and Its Interactions with Nisin. *Journal of Biological Chemistry* **290,** 28869–28886 (2015).
7. Richter, C. D., Nietlispach, D., Broadhurst, R. W. & Weissman, K. J. Multienzyme docking in hybrid megasynthetases. *Nat Chem Biol* **4,** 75–81 (2008).
8. Dowling, D. P. *et al.* Structural elements of an NRPS cyclization domain and its intermodule docking domain. *Proc. Natl. Acad. Sci. U.S.A.* **113,** 12432–12437 (2016).
9. Kelly, S. M. & Price, N. C. The use of circular dichroism in the investigation of protein structure and function. *Curr. Protein Pept. Sci.* **1,** 349–384 (2000).
